# Sleep deprivation induces anxiety-like behaviors through IL-6 driven astrocyte-GABAergic neuron crosstalk in the PAG-ACC circuit

**DOI:** 10.1186/s12974-026-03879-z

**Published:** 2026-05-22

**Authors:** Xiao Xu, Ziang Wu, Wenxin Hang, Shaojia Mao, Jiahui Sun, Huanbin Xiong, Jinpiao Zhu, Daqing Ma

**Affiliations:** 1 Perioperative and Systems Medicine Laboratory, Department of Rehabilitation, Department of Anesthesiology, Children’s Hospital, National Clinical Research Center for Children and Adolescents’ Health and Diseases, Zhejiang University School of Medicine, Hangzhou, 310015 China; 2 Department of Anesthesiology, The First Affiliated Hospital, Zhejiang University School of Medicine, Hangzhou, 310003 Zhejiang China; 3 Department of Anesthesiology, Zhongnan Hospital, Wuhan University, Wuhan, 430071 China; 4 Division of Anesthetics, Pain Medicine & Intensive Care, Department of Surgery and Cancer, Faculty of Medicine, Imperial College London, Chelsea and Westminster Hospital, London, SW10 9NH UK

**Keywords:** Sleep deprivation, Anxiety, Pro-inflammatory cytokine, IL-6, Periaqueductal gray, Mesencephalic astrocyte-derived neurotrophic factor

## Abstract

Sleep deprivation (SD) is associated with anxiety-like behaviors, yet the underlying molecular and neural circuit mechanisms remain unclear. Using a 24-hour SD paradigm in mice, we found that SD induced anxiety-like behaviors and increased interleukin-6 (IL-6) in blood and the periaqueductal gray (PAG), and PAG-targeted administration of IL-6 receptor antagonist rescued the anxiety-like behaviors. This increased IL-6 activated PAG astrocytes, as evidenced by morphological remodeling, increased calcium activity, and enhanced glutamate release. Astrocytic activation upregulated several genes associated with endoplasmic reticulum (ER) protein synthesis and processing, including neuronal mesencephalic astrocyte-derived neurotrophic factor (*Manf*). Knockdown of *Manf* in PAG neurons upregulated the GABA_A_ receptor α1 subunit (Gabra1), reduced the activity of GABAergic neurons projecting to the anterior cingulate cortex (ACC), and rescued anxiety-like behaviors. Chemogenetic inhibition of this PAG-ACC circuit prevented SD-induced anxiety-like behaviors, whereas its activation recapitulated the anxiety-like behaviors. Our study identifies an IL-6-astrocyte-Manf signaling axis in the PAG that drives anxiety-like behaviors *via* enhanced GABAergic neuronal output to the ACC. This PAG-ACC circuit may serve as a neuroimmune hub for SD-mediated anxiety-like behaviors and represents a potential therapeutic target.

**Supplementary Information:**

The online version contains supplementary material available at 10.1186/s12974-026-03879-z.

## Introduction

Sleep is an indispensable physiological process and crucial for cognition and memory consolidation through maintenance of physiological metabolic homeostasis and normal synaptic function [1, 2]. As such, sleep disorders negating people’s life quality is enormous and is emerging as a significant public health burden worldwide [3]. Accumulating evidence shows inadequate sleep to be an increased risk of diverse detrimental outcomes, including cardiovascular dysfunction, metabolic syndrome, and compromised immune function [4–6]. Among these wide-ranging complications, the association between sleep disturbance and adverse emotional states, particularly depression and anxiety, is of clinical significance [7, 8]. Although this association is evident in clinic [9, 10], the underlying neurobiological mechanisms remain to be elucidated.

The brain regions, e.g., the lateral habenula (LHb), paraventricular nucleus of the hypothalamus (PVN), and amygdala play important roles in responsible for negative emotional behaviors [11, 12]. Notably, the periaqueductal gray (PAG), traditionally recognized for its roles in social hierarchy and pain processing, has gained increasing attention for its emerging functions in sleep-wake regulation and emotional processing [13–16]. Previous studies showed that PAG GABAergic neurons strongly suppressed rapid eye movement (REM) sleep onset and activated its termination promoted non-rapid eye movement (NREM) sleep [17, 18]. However, it remains unknown whether sleep deprivation alters the activity of PAG GABAergic neurons and whether such changes contribute to the emergence of negative emotional states following deprived sleep.

A hallmark of sleep deprivation is systemically inflammatory, which is commonly characterized by a surge of pro-inflammatory cytokines, including IL-6 and TNF-α [19]. This peripheral immune activation can communicate with the central nervous system, contributing to neuroinflammation, which is increasingly recognized to be detrimental on emotion and cognition [20, 21]. A previous study demonstrated that increased inflammatory cytokines in the PAG promote pain behaviors, and that pharmacological inhibition of their receptors mitigates these effects [22]. However, whether sleep deprivation similarly alters cytokine levels in the PAG, and whether such neuroimmune changes contribute to the negative emotional consequences of sleep deprivation is unclear.

Therefore, we used multidisciplinary approaches including behavioral tests, fiber photometry, neural circuit tracing, and chemogenetic manipulations to investigate underlying mechanisms of sleep deprivation associated negative emotion. We found that sleep deprivation triggers a specific increase of IL-6 both systemically and within the midbrain, leading to the activation of astrocytes in the PAG. These activated astrocytes drive the upregulation of mesencephalic astrocyte-derived neurotrophic factor (MANF) in PAG neurons, which subsequently suppresses GABA_A_ α1 receptor expression, and disinhibits PAG GABAergic neuronal projection to the anterior cingulate cortex (ACC), ultimately results in anxiety-like behaviors.

## Materials and methods

### Mice

All experiments were performed in strict accordance with the ARRIVE Guideline for the Care and Use of Laboratory Animals, and the protocols were approved by the Animal Ethics Committee of Zhejiang University (NO.35095). 8-week-old male C57BL/6J mice were obtained from Hangzhou Enlighten the Truth Laboratory Animal Technology Co., Ltd (Hangzhou, Zhejiang, China) were housed (3–5 mice/cage) in a climate-controlled room (temperature: 22 ± 1 °C; humidity: 50 ± 10%) with a 12-hour light/dark cycle (lights on at 7 a.m. and lights off at 7 p.m.). Animals were free access to food and water. For microglial depletion, mice were fed with a diet containing PLX5622 (1200 ppm, M18090601, Moldiets, China) for three consecutive weeks prior to experiments.

### Sleep deprivation

Sleep deprivation (SD) was performed using a horizontal rotating rod apparatus. The SD period was conducted for 24 h from 8 a.m. to the next day of 8 a.m. The rotation speed was set at 3 revolutions/minute (rpm), with a frequency of 10-second rotated period followed by a 20-second interval as reported previously [23].

### EEG/EMG recoding and sleep-wake state analysis

Mice were anesthetized with 2% isoflurane, and then fixed on a digital stereotaxic instrument (68018, RWD Life Science, China) for electroencephalogram (EEG)/ electromyography (EMG) electrodes implantation. EEG electrodes were placed epidurally over the right and left sides of frontal (anterior-posterior (AP): +1.5 mm, medial-lateral (ML): -1.5 mm) and parietal (AP: -3.0 mm, ML: -2.5 mm) cortices. Two insulated wires for EMG were inserted into the nuchal muscle group to record neck muscle activity. All electrodes were secured to the skull using dental acrylic cement.

After a 7-day post-surgery period, mice were habituated to the EEG/EMG recording chambers for 24 h. Continuous EEG/EMG signals were then recorded for 24 h under a standard 12-hour light/dark cycle. Signals were amplified, filtered and digitized at a sampling rate of 1000 Hz using the Medusa small animal electrophysiology recording system (Medusa, Bio-Signal Technologies, China). According to our previous studies [24, 25], EEG/EMG signals were analyzed using a semi-automated sleep-scoring software. Sleep-wake states were classified into 10-second epochs as wakefulness, non-rapid eye movement (NREM) sleep and rapid eye movement (REM) sleep. Total time spent in wakefulness, NREM sleep, and REM sleep, latency to sleep onset, and the number and duration of individual sleep bout were quantified.

### Open Field Test (OFT)

All behavioral tests were conducted during day-time. Mice were acclimatized to the test room for at least 60 min prior to the start of each test. For OFT, each mouse was gently placed in the center of a square-shaped arena (40 cm × 40 cm × 40 cm) and allowed to explore freely for 10 min. Their movement was recorded by a video camera mounted above the arena. The total distance traveled was quantified to evaluate locomotor activity. Anxiety-like behavior was measured as the time spent in the center zone, and the number of entries into the center zone.

### Elevated Plus Maze (EPM) test

Anxiety-like behavior was further evaluated using the EPM test. The apparatus consisted of two open arms (30 cm × 5 cm) and two enclosed arms (30 cm × 5 cm × 15 cm) extending from a central platform (5 cm × 5 cm), 50 cm above the floor. Each mouse was placed at the center of the maze, facing an open arm, and allowed to explore for 10 min. The number of entries into the open arms and the time spent in the open arms were recorded.

### Enzyme-linked immunosorbent assay

After anesthesia with 2% isoflurane, blood (1 mL) *via* the inferior vena cava and brain tissue were collected. The blood samples were left to stand for 30 min and then centrifuged at 3500 rpm for 10 min to obtain serum. Serum levels of pro-inflammatory cytokines including IL-1β, IL-6 and TNF-α were measured using enzyme-linked immunosorbent assay (ELISA) kits (IL-1β: EM0029, Hua’an Biotechnology Co., Ltd, China; IL-6: EM0004, Hua’an Biotechnology Co., Ltd, China; TNF-α: EM0010, Hua’an Biotechnology Co., Ltd, China). The assay was performed by adding 20 µL serum per well, with each sample measured in triplicate, then followed the manufacturer’s protocol.

### Real-time quantitative polymerase chain reaction

Following homogenization of brain tissues in TRIzol reagent (15596018CN, Invitrogen, USA), total RNA was isolated using the manufacturer’s recommended procedure. To minimize degradation, the purified RNA was promptly reverse-transcribed into cDNA (R233-01, Vazyme, China). Quantitative real-time PCR (qPCR) assays (Q711; Vazyme, China) were carried out using the cDNA templates and the primer sequences provided in Table S1. The relative quantification of target gene expression was analyzed by the comparative threshold cycle (2^^−ΔΔCt^) method.

### Intravenous IL-6 administration

Recombinant Interleukin 6 (IL-6) (IL-6-H8218, Acrobiosystems, China) was diluted in sterile saline. The indicated dose (2ug/ml) was administered *via* the lateral tail vein in a total volume of 100 µL. Control mice received an equal volume of saline. The injection was performed under gentle manual restraint without anesthesia.

### Micro-infusion

Under surgical anaesthesia, guided cannulas (4 mm in length) were implanted targeting the periaqueductal gray (PAG) and the anterior cingulate cortex (ACC) under aseptic conditions. The stereotaxic coordinates for the PAG and ACC were (PAG: AP: -4.23 mm, ML: ± 0.4 mm, DV: − 2.5 mm; ACC: AP: +1.1 mm, ML: ±0.25 mm, DV: -1.65 mm). The cannulas were secured with dental acrylic cement. Following a 7-day recovery, the solution of IL-6 receptor antagonist (IL-6Ra) (HY-P10284, MedChemExpress, USA), GABA receptor antagonist (GABARa) (HY-W008645, MedChemExpress, USA), NMDA receptor antagonist AP5 (HY-100714 A, MedChemExpress, USA), AMPA receptor antagonist CNQX (HY-15066, MedChemExpress, USA) or vehicle was administered using a micro-infusion pump connected to an internal injector (R462, RWD Life Science, China) that extended 0.5 mm beyond the tip of the guide cannula. A total volume of 0.3 µL per site was infused at a constant rate of 0.1 µL/min. The injector was left in place for an additional 5 min after infusion to ensure the complete diffusion of the solution and minimize backflow.

### Adeno-associated virus (AAVs) injection

All adeno-associated viruses (AAVs) used in this study were commercially obtained. Their specific details, including serotype, promoter, transgene, and titer, were provided in Table S2. Mice were anesthetized with 2% isoflurane, and securely placed in a stereotaxic frame. A heating pad was used to maintain body temperature at 37 °C throughout the surgery. The scalp was shaved and disinfected alternately with betadine and 75% ethanol three times.

A midline incision was made along the scalp to expose the skull. The skull surface was cleaned and dried. The adeno-associated virus (AAV) (titer > 1 × 10¹² vg/mL) was loaded into a glass micropipette. A small craniotomy was drilled above each target coordinate. The injection needle was slowly lowered to the target depth at a rate of 200 μm/minute. The virus was infused using a microinjection pump (R-480, RWD Life Science, China) at a controlled rate of 30 nL/minute. The injection volume for each site was 200 nL. When the infusion was complete, the needle was left for an additional 7–10 min to allow for complete diffusion of the virus and to prevent backflow upon withdrawal. The scalp incision was sutured and treated with topical antibiotic ointment. The mouse was placed in a clean, warm cage for recovery. Analgesics (Meloxicam, 5 mg/kg, HY-B0261, MCE, China) were administered subcutaneously for post-operative pain relief for 2–3 consecutive days when necessary.

### Fiber photometry

An AAV encoding the astrocyte-specific calcium indicator (AAV2/5-GfaABC1D-GCaMP6s) or the glutamate sensor (AAV2/9-hSyn-iGluSnFR) was injected into the PAG, or an AAV for GABA sensor iGABASnFR was injected into the ACC. Subsequently, an optical fiber (230 μm in diameter, Xi’an Bogao Optoelectronic Technology Co., Ltd., China) was implanted 0.1 mm above the PAG or ACC. The scalp incision was then closed with dental cement. After 3-week post-surgery, calcium activity of PAG astrocytes or dynamic glutamate/GABA release were recorded during EPM test using a fiber photometry system (Thinker Tech Nanjing Co., Ltd., China). The signals were analyzed using offline software. The relative change in fluorescence was calculated as *ΔF (F-F*_*0*_*)/F*_*0*_, where *F*_*0*_ represented the baseline fluorescence, averaged over a 5 s time widow before entry into the open arms.

### Immunohistochemistry

Mice were deeply anesthetized, and transcardially perfused with 30 mL of phosphate-buffered saline (PBS), followed by 30 mL of 4% paraformaldehyde (BL539A, Biosharp, China) in PBS solution for fixation. The brains were then carefully dissected and post-fixed in 4% PFA at 4 °C overnight. Subsequently, the brains were cryoprotected by immersion in 30% sucrose solution at 4 °C until they sank the bottom. The cryoprotected brains were embedded in optimal cutting temperature compound (OCT; 6502, Epredia, USA) and coronally sectioned into 30-µm-thick slices using a cryostat (CryoStar NX70, Thermo Fisher Scientific, USA). The sections were permeabilized with 0.3% Triton X-100 (ST1723, Beyotime Biotechnology, China) in PBS for 30 min at room temperature. Then, they were blocked with blocking buffer (P0260, Beyotime Biotechnology, China) for 2 h at room temperature. Subsequently, the sections were incubated with primary antibodies (Anti-IL-6, 1:100, ab290735, abcam, UK; Anti-Iba1, 1:500, OB-PGP049-01, OasisBiofarm, China; Anti-c-Fos, 1:1000, ab190289, abcam, UK; Anti-c-Fos, 1:1000, 226308, Synaptic Systems, Germany; Anti-GABA, 1:400, A2052, Sigma, USA; Anti-GFAP, 1:400, 12389, Cell Signaling Technology, USA; Anti-NeuN, 1:1000, ab104224, abcam, UK; Anti-MANF, 1:400, ab316835, abcam, UK; Anti-GABRA1, 1:400, ab252430, abcam, UK) diluted in antibody dilution buffer (P0103, Beyotime Biotechnology, China) at 4 °C overnight. The sections were then washed three times with PBS (5 min per wash). This was followed by incubation with fluorophore-conjugated secondary antibodies (Alexa Fluor 488-conjugated Goat anti-Rabbit, 1:400, 111-545-003, Jackson ImmunoResearch Inc., USA; Alexa Fluor 488-conjugated Goat anti-Mouse, 1:400, 115-545-003, Jackson ImmunoResearch Inc., USA; Alexa Fluor 488-conjugated Goat anti-Guinea Pig, 1:400, 106-545-003, Jackson ImmunoResearch Inc., USA; Alexa Fluor 555-conjugated Goat anti-Rabbit, 1:400, 111-565-144, Jackson ImmunoResearch Inc., USA) for 2 h at room temperature protected from light. After incubation, the sections were washed three times with PBS (10 min per wash). Finally, the sections were mounted with an anti-fade mounting medium and coverslipped. The stained sections were imaged using a confocal laser scanning microscope (Leica-sp8, Leica, Germany). Quantitative analysis of fluorescence intensity was performed with ImageJ software (version:1.53q, National Institutes of Health, USA).

### Western blot

Mice were anesthetized with 2% isoflurane, and subjected to transcardial perfusion with PBS. The brains were then rapidly removed and sectioned into coronal slices. The periaqueductal gray (PAG) region was meticulously microdissected from these slices. The PAG tissues were immediately homogenized in RIPA lysis buffer (P0013B, Beyotime Biotechnology, China) supplemented with protease and phosphatase inhibitors (P1005, Beyotime Biotechnology, China). The homogenates were incubated on ice for 1 h to ensure complete lysis, followed by centrifugation at 12,000 rpm for 10 min at 4 °C.

The protein concentration of each lysate was determined using a BCA protein assay kit (P0012S, Beyotime Biotechnology, China) according to the manufacturer’s instructions. Equal amounts of protein (20 µg) from each sample were separated by sodium dodecyl sulfate-polyacrylamide gel electrophoresis (Mini-PROTEAN Tetra, Bio-Rad, USA) and subsequently transferred onto polyvinylidene fluoride (PVDF) membranes (IPVH00010, Millipore, USA). The membranes were blocked with 5% non-fat milk in TBST (Tris-buffered saline with 0.1% Tween-20) for 1 h at room temperature and then incubated with primary antibodies (Anti-MANF, 1:1000, ab316835, abcam, UK; Anti-GABRA1, 1:1000, ab252430, abcam, UK) diluted in blocking buffer overnight at 4 °C. After washing with TBST, the membranes were incubated with appropriate horseradish peroxidase (HRP)-conjugated secondary antibodies (SL1350, Coolaber, China) for 1 h at room temperature. Protein bands were visualized using an enhanced chemiluminescence detection system (ChemiDoc MP, Bio-Rad, USA). ImageJ software was used for the analysis of the band intensity (version:1.53q, National Institutes of Health, USA).

### Bulk RNA-Sequencing and bioinformatics analysis

Mice were anesthetized with 2% isoflurane and transcardially perfused with RNase-free PBS to remove blood cells and minimize background RNA. The brains were rapidly extracted and sectioned into coronal slices. PAG region was precisely microdissected from the slices. Then, the collected PAG tissues were immediately snap-frozen in liquid nitrogen and stored at -80 °C until RNA extraction. Total RNA was extracted using the TRIzol reagent (Invitrogen, USA) according to the manufacturer’s protocol. RNA purity and quantification were evaluated using the NanoDrop 2000 spectrophotometer (Thermo Fisher Scientific, USA). RNA integrity was assessed using the Agilent 2100 Bioanalyzer (Agilent Technologies, USA). Then the libraries were constructed using VAHTS Universal V10 RNA-seq Library Prep Kit (Premixed Version) according to the manufacturer’s instructions. The transcriptome sequencing and analysis were conducted by OE Biotech Co., Ltd. (Shanghai, China).

The libraries were sequenced on an Illumina Novaseq 6000 platform and 150 bp paired-end reads were generated. Raw reads of fastq format were firstly processed using fastp and the low quality reads were removed to obtain the clean reads. The clean reads were mapped to the referenced genome using HISAT2. FPKM of each gene was calculated and the read counts of each gene were obtained by HTSeq-count. PCA analysis were performed using R (v 3.2.0) to evaluate the biological duplication of samples. Differential expression analysis was performed using the DESeq2. Adjusted p-value (padj) < 0.05 and |log2(Fold Change) | > 0.58 was set as the threshold for significantly differential expression gene (DEGs). GO, KEGG pathway, Reactome and WikiPathways enrichment analysis of DEGs were performed to screen the significant enriched term using R (v 3.2.0). Gene Set Enrichment Analysis (GSEA) was conducted to evaluate the distribution of predefined gene sets within the ranked list of all genes based on their differential expression using R (v 3.2.0).

### Statistical analysis

The investigators were blinded during the group allocation, data acquisition, and subsequent statistical analysis. All data were presented as the mean ± standard error of the mean (SEM). Statistical analyses were performed using GraphPad Prism (version: 9.5.0; GraphPad Software, USA). For comparisons between two groups, data normality was assessed using the Shapiro-Wilk test and homogeneity of variances was assessed using the F-test. Accordingly, Student’s unpaired two-tailed t-test (for normal data with equal variances), Welch’s t-test (for normal data with unequal variances), or the Mann-Whitney U test (for non-normal data) was applied where appropriate. For repeated measures data, two-way repeated measures (RM) ANOVA was conducted, followed by Fisher’s LSD or Šidák’s multiple comparisons test. Statistical significance was defined as *p* < 0.05.

## Results

### Sleep deprivation induced anxiety-like behaviors

**Fig. 1:**
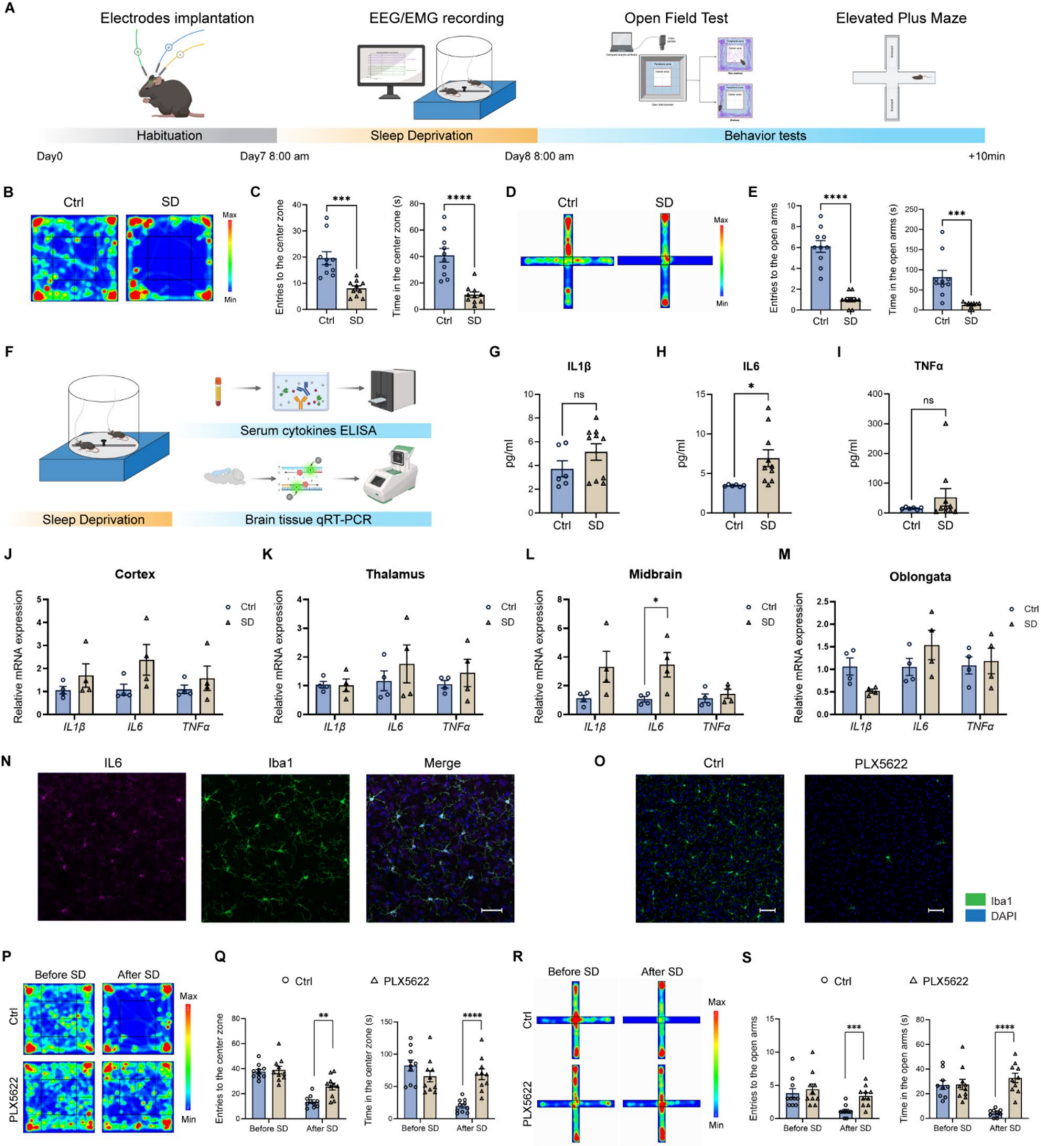
Sleep deprivation induced anxiety-like behaviors and increased pro-inflammatory cytokine IL-6 in blood and midbrain. **A** Schematic diagram of the behavioral experimental paradigm. **B** Representative heatmaps of mouse movement in the open field test (OFT) in the control group and the sleep deprivation (SD) group. **C** The number of entries into the center zone and the time spent in the center zone during the OFT (*n* = 10). **D** Representative heatmaps of mouse movement in the elevated plus maze (EPM) test in the control group and the SD group. **E** The number of entries into the open arms and the time spent in the open arms during the EPM test (*n* = 10). **F** Flowchart illustrating the timeline for tissue collection. **G-I** Serum concentrations of pro-inflammatory cytokines IL-1β, IL-6, and TNF-α in the control group (*n* = 6) and the SD group (*n* = 10). **J-M** mRNA expression levels of *IL-1β*, *IL-6*, and *TNF-α* in the cerebral cortex, thalamus, midbrain and oblongata in both groups (*n* = 4). **N** Representative immunofluorescence images showing co‑localization of Iba1 and IL‑6 in the midbrain after sleep deprivation (SD). Scale bar:50 μm. **O** Representative immunofluorescence images of Iba1 expression after treatment with PLX5622 or control diet. Scale bar: 50 μm. **P** Representative heatmaps of mouse movement in the OFT in the control group and the PLX5622‑treated group. **Q** The number of entries into the center zone and the time spent in the center zone during the OFT (*n* = 10). **R** Representative heatmaps of mouse movement in the EPM test in the control group and the PLX5622‑treated group. **S** The number of entries into the open arms and the time spent in the open arms during the EPM test (*n* = 10). Data were presented as mean ± SEM; *p < 0.05, **p < 0.01, ***p < 0.001, ****p < 0.0001

To investigate the effects of sleep deprivation (SD) on anxiety-like behaviors, we firstly established a mouse model of acute sleep deprivation using a horizontal rotating rod apparatus that we reported previously [26]. Following a one-week recovery from electroencephalography (EEG) and electromyography (EMG) electrodes implantation under anaesthesia (Fig. 1A), the baseline of 24-hrs sleep-wake cycles from 8 am (ZT 0) to the next day of 8 am (ZT 24) were monitored. They were then subjected to SD paradigm (Fig. 1A). Concurrent EEG/EMG recordings showed an increase in total duration of wakefulness state (13.65 ± 0.42 vs. 11.30 ± 0.23 h of the Ctrl group, t(6) = 4.221, *p* = 0.0056) and a decrease in total duration of rapid eye movement (REM) sleep (0.86 ± 0.26 vs. 2.11 ± 0.12 h of the Ctrl group, t(6) = 3.845, *p* = 0.0085) in SD mice, but not in non-rapid REM (NREM) sleep, compared to controls (Figure S1A-F).

We next assessed the anxiety-like behaviors with the open field test (OFT) and elevated plus maze (EPM) test (Fig. 1A). In the OFT, SD mice showed a decrease in both the number of entries into the center zone (8.00 ± 0.93 vs. 19.60 ± 2.35 of the Ctrl group, t(18) = 4.355, *p* = 0.0004) and the time spent in the center zone (11.07 ± 2.06 vs. 40.92 ± 4.90 of the Ctrl group, t(18) = 5.331, *p* = 0.0007) (Fig. 1B-C). Similarly, in the EPM test, SD mice spent significantly less time in the open arms (12.98 ± 2.25 vs. 81.79 ± 15.76 of the Ctrl group, t(18) = 4.1, *p* < 0.0001) and crossed fewer entries into the open arms (1.00 ± 0.20 vs. 6.10 ± 0.54 of the Ctrl group, t(18) = 8.435, *p* < 0.0001) (Fig. 1D-E).

### Sleep deprivation increased peripheral and midbrain pro-inflammatory cytokine IL-6

To determine whether sleep deprivation (SD) triggers a systemic inflammatory response, we measured serum pro-inflammatory cytokine levels including interleukin-1 beta (IL-1β), interleukin-6 (IL-6) and tumor necrosis factor-alpha (TNF-α). The serum levels of IL-6 were increased (6.93 ± 1.01 vs. 3.47 ± 0.04 of the Ctrl group, t(14) = 2.485, *p* = 0.0262) in SD mice compared to control ones, but not in IL-1β or TNF-α (Fig. 1G-I). Furthermore, we quantified mRNA expression of these cytokines in discrete brain regions including cortex, thalamus, midbrain and oblongata. An increase in *IL-6* expression in the midbrain (3.47 ± 0.74 vs. 1.06 ± 0.16 of the Ctrl group, t(6) = 2.751, *p* = 0.0332), with no significant changes in the cortex, thalamus, or oblongata (Fig. 1J-M).

### Microglia-derived IL‑6 contributed to the anxiety‑like behaviors after sleep deprivation

To determine the cellular source of elevated IL‑6 after sleep deprivation (SD), we performed immunofluorescence co‑staining IL‑6 and the microglial marker Iba1 in the midbrain. As shown in Fig. 1N, IL‑6 was predominantly co‑localized with the Iba1‑positive microglia, suggesting that microglia are a major source of SD‑induced IL‑6. Furthermore, we depleted microglia by feeding mice with PLX5622 (microglia depletion)‑containing diet followed by SD. PLX5622 treatment attenuated SD‑induced IL‑6 expression (1.46 ± 0.65 vs. 2.49 ± 1.11 of the Ctrl group, t(8) = 6.555, *p* = 0.0002) (Figure S1G-H). Moreover, SD mice treated with PLX5622 exhibited improved anxiety‑like behaviors, as evidenced by increased entries into the center zone (25.70 ± 2.57 vs. 13.70 ± 1.43 of the Ctrl group, t(18) = 3.868, *p* = 0.0011) and time spent in the center zone (68.85 ± 7.85 vs. 19.79 ± 3.02 of the Ctrl group, t(18) = 5.531, *p* < 0.0001) in the open field test (OFT), and by increased entries into the open arms (3.40 ± 0.45 vs. 1.10 ± 0.26 of the Ctrl group, t(18) = 4.176, *p* = 0.0006) and time spent in the open arms (32.83 ± 3.59 vs. 4.27 ± 0.88 of the Ctrl group, t(18) = 7.334, *p* < 0.0001) in the elevated plus maze (EPM) test (Fig. 1P‑S). These data collectively demonstrated that microglia are the primary cellular source of IL‑6 elevation after SD, and that microglia‑derived IL‑6 critically contributes to the anxiogenic effects of SD.

### IL-6 signaling in the PAG was involved in anxiety-like behaviors induced by sleep deprivation

**Fig. 2:**
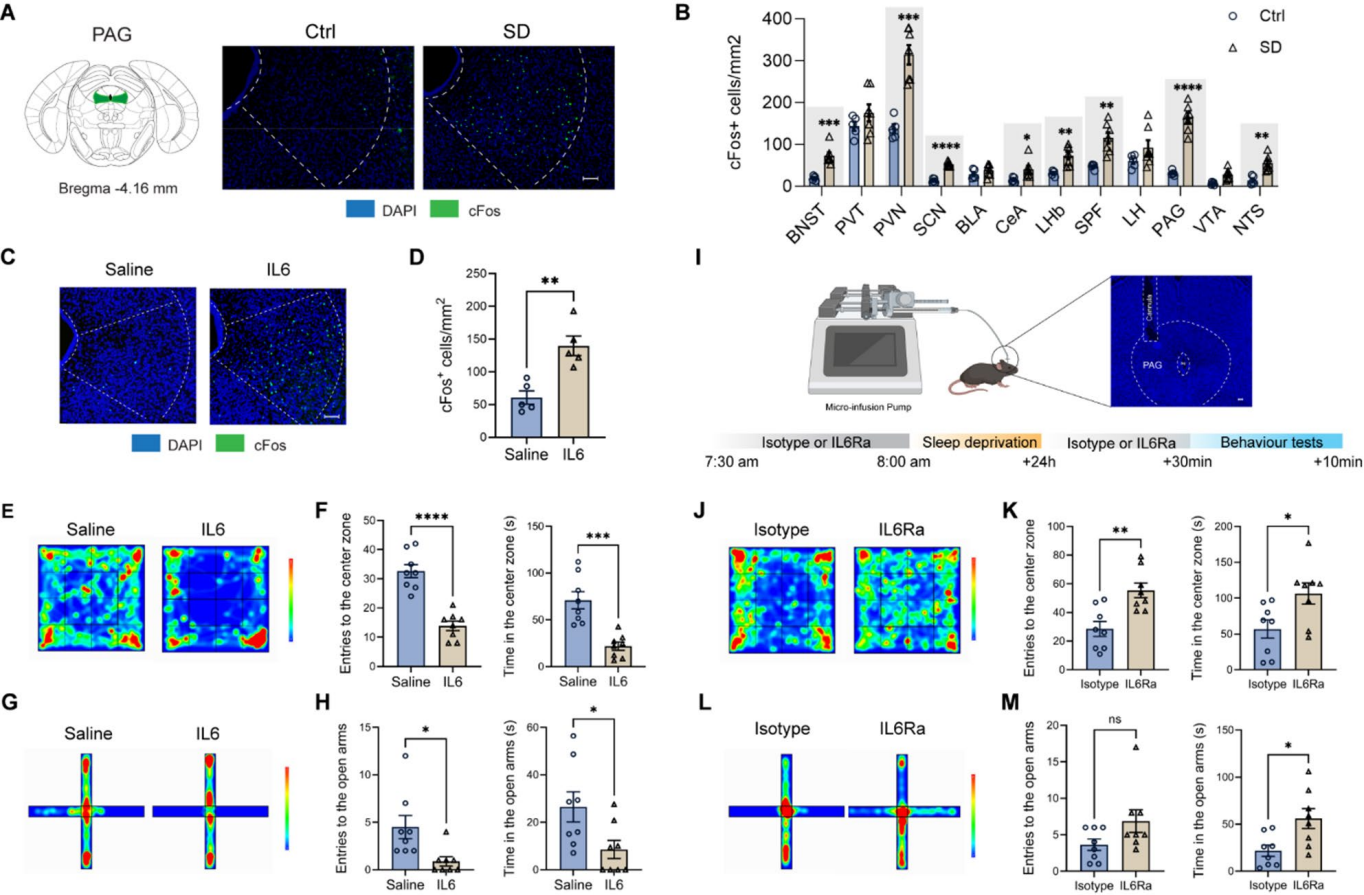
IL-6- signaling Inhibition in the PAG rescued SD-induced anxiety-like behaviors. **A** Representative immunofluorescence images showing c-Fos expression in the periaqueductal gray (PAG) in the control group and the sleep deprivation (SD) group. Scale bar: 100 μm. **B** Quantification of c-Fos-positive cells in the PAG and other brain regions following SD. **C** Representative immunofluorescence images of c-Fos expression in the PAG 3 h after intravenous injection of saline or IL-6. **D** Quantitative analysis of c-Fos-positive cells in the PAG after saline or IL-6 treatment (*n* = 5). **E** Representative heatmaps of mouse movement in the open field test (OFT) 3 h after saline or IL-6 treatment. **F** The number of entries into the center zone and the total time spent in the center zone during the OFT (*n* = 8). **G** Representative heatmaps of mouse movement in the elevated plus maze (EPM) test 3 h after saline or IL-6 treatment. **H** The number of entries into the open arms and the total time spent in the open arms during the EPM test (*n* = 8). **I** Schematic diagram and timeline of the drug micro-infusion into the PAG, and a representative image verifying the cannula placement. **J** Representative heatmaps of movement tracing in the OFT following micro-infusion of an IL-6 receptor antagonist or its isotype control. **K** The number of entries into center zone and the total time spent in the center zone (*n* = 8). **L** Representative heatmaps of movement tracing in the EPM test following micro-infusion of the IL-6 receptor antagonist or isotype control. **M** The number of entries into the open arms and the total time spent in the open arms (*n* = 8). Data were presented as mean ± SEM; *p < 0.05, **p < 0.01, ***p < 0.001, ****p < 0.0001

To identify brain regions activated by sleep deprivation (SD), we mapped whole-brain neuronal activity using c-Fos immunostaining, and found increased number of c-Fos positive (c-Fos^+^) cells in the brain regions associated with stress and emotional processing, including the bed nucleus of the stria terminalis (BNST), paraventricular nucleus of the hypothalamus (PVN), central amygdala (CeA), and the periaqueductal gray (PAG) (Fig. 2A-B; Figure S2).

Given that SD upregulated IL-6 levels in both serum and midbrain, we hypothesized that IL-6 might mediate anxiety-like behaviors induced by SD. Intravenous administration of IL-6 to control mice selectively increased the number of c-Fos^+^ cells in the PAG (139.80 ± 13.34 vs. 60.80 ± 8.99 of the Saline group, t(8) = 4.394, *p* = 0.0023) (Fig. 2C-D). Furthermore, mice treated with IL-6 demonstrated the anxiety-like phenotype, as evidence by a decrease in center-zone entries (13.88 ± 1.51 vs. 32.63 ± 2.14 of the Saline group, t(14) = 6.699, *p* < 0.0001) and time spent in the center zone (21.84 ± 4.06 vs. 70.91 ± 8.60 of the Saline group, t(14) = 4.829, *p* = 0.0003) in the open field test (OFT) (Fig. 2E-F), as well as a reduction in open-arm entries (0.88 ± 0.45 vs. 4.50 ± 1.15 of the Saline group, t(14) = 2.756, *p* = 0.0155) and time spent in open arms (8.51 ± 3.56 vs. 26.50 ± 5.95 of the Saline group, t(14) = 2.427, *p* = 0.0293) in the elevated plus maze (EPM) test (Fig. 2G-H).

To further examine the role of IL-6 signaling in SD-induced anxiety-like behaviors, we locally administered an IL-6 receptor antagonist (IL-6Ra) or an isotype control directly into the PAG before and during SD to further investigate IL-6 signaling role in anxiety-like behaviors induced by SD (Fig. 2I). Blockade of IL-6 signaling in the PAG rescued SD-induced anxiety-like behaviors as showing increased the number of center-zone entries (55.38 ± 4.86 vs. 28.50 ± 4.86 of the Isotype group, t(14) = 3.656, *p* = 0.0026) and time spent in the center zone (106.48 ± 13.86 vs. 56.79 ± 11.93 of the Isotype group, t(14) = 2.542, *p* = 0.0235) in the OFT (Fig. 2J-K) as well as increased number of entries into the open arms (6.88 ± 1.47 vs. 3.63 ± 0.73 of the Isotype group, t(14) = 1.85, *p* = 0.0855) and increased time spent in the open arms (55.94 ± 9.97 vs. 21.76 ± 5.55 of the Isotype group, t(14) = 2.802, *p* = 0.0141, Fig. 2L-M) of the EPM test in the IL-6Ra–treated mice compared to the isotype controls following SD.

### Astrocytic activation in the PAG contributed to anxiety-like behaviors

**Fig. 3:**
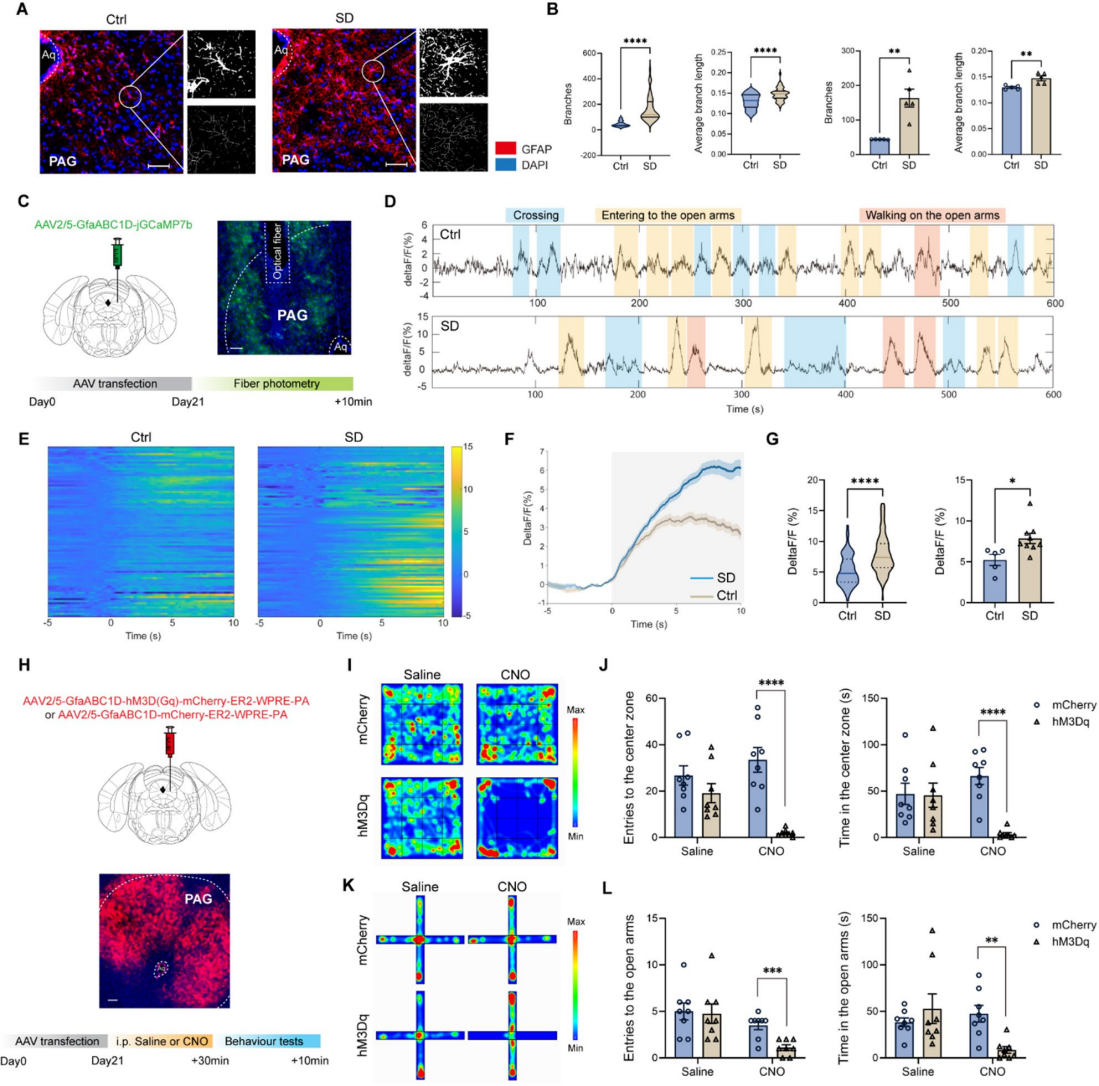
PAG activated astrocytes contributed to sleep deprivation-induced anxiety-like behaviors. **A** Representative immunofluorescence images of glial fibrillary acidic protein (GFAP) expression in the periaqueductal gray (PAG) of the control group and the sleep deprivation (SD) group. Scale bar: 50 μm. **B** Quantification of astrocytic morphology, including the number of branches and total branch length, in the PAG following SD (*n* = 5). **C** Schematic of the virus injection into the PAG for fiber photometry, and a representative image verifying the fiber placement. **D** Representative traces of PAG astrocytic calcium activity during the elevated plus maze (EPM) test in both groups. Shaded areas represented the time spent in the open arms. **E** Heatmap showing the astrocytic calcium signals during the EPM test, aligned to the entries into the open arms. **F** Time-course curve of the average astrocytic calcium activity during the entries into the open arms in the EPM test. **G** Quantification of the peak *ΔF/F* of astrocytic calcium signals during the entries into the open arms in the EPM test in both groups (*n* = 5 for the control group and *n* = 9 for the SD group). **H** Schematic of the chemogenetic activation of PAG astrocytes, and a representative image verifying the viral expression. **I** Representative heatmaps of movement tracing in the open field test (OFT) following chemogenetic activation of PAG astrocytes. **J** The number of entries into the center zone and the total time spent in the center zone during the OFT (*n* = 8). **K** Representative heatmaps of movement tracing in the EPM test following chemogenetic activation of PAG astrocytes. **L** The number of entries into the open arms and the total time spent in the open arms during the EPM test (*n* = 8). Data were presented as mean ± SEM; *p < 0.05, **p < 0.01, ***p < 0.001, ****p < 0.0001

Given the susceptibility of astrocytes to immunological stimuli and their essential roles in the support of neuronal function, we asked whether they contribute to IL-6-driven anxiety associated with the periaqueductal gray (PAG). Morphological remodeling [27] and increased calcium activity [28] are hallmarks of astrocyte activation, we further analyzed GFAP-based morphological changes and calcium activity evoked by anxiety-provoking behaviors following sleep deprivation (SD). Immunostaining for the marker of astrocytes, glial fibrillary acidic protein (GFAP), showed that SD induced astrocyte hypertrophy in the PAG, including an increase in both branch number (163.24 ± 23.11 vs. 43.86 ± 0.29 of the Ctrl group, t (8) = 4.62, *p* = 0.0017) and length (0.1475 ± 0.0041 vs. 0.1298 ± 0.0016 of the Ctrl group, t (8) = 3.563, *p* = 0.0074) (Fig. 3A-B). Furthermore, we monitored calcium dynamics of PAG astrocytes using in-vivo fiber photometry during the elevated plus maze (EPM) test. In control mice, the anxiety-provoking events, e.g., transition from the closed arms to the open arms and walking in the open arms, induced an increased calcium activity of astrocytes (Fig. 3D-G). Moreover, SD further increased this behaviorally-correlated activity (7.87 ± 0.58 vs. 5.23 ± 0.60 of the Ctrl group, t (12) = 2.705, *p* = 0.0191) (Fig. 3D-G).

Having established that SD enhances astrocytic activation, we next investigated whether IL‑6 is sufficient to drive these changes. Intravenous administration of exogenous IL‑6 recapitulated the augmented calcium activity in PAG astrocytes during the EPM test (10.06 ± 0.69 vs. 5.96 ± 0.51 of the Saline group, t (6) = 4.643, *p* < 0.0001; Figure S3A–D). Conversely, microglia depletion using PLX5622 not only reduced SD‑induced IL‑6 levels but also prevented PAG astrocytic hypertrophy, as reflected by a marked reduction in both the number of branches (25.20 ± 1.34 vs. 104.85 ± 17.20, t(38) = 4.500, *p* < 0.0001) and average branch length (0.1279 ± 0.0027 vs. 0.2295 ± 0.0113, t(38) = 8.495, *p* < 0.0001) compared to control diet-fed mice (Figure S3E-F), suggesting that IL-6 dysregulation contributes to astrocyte activation following SD.

Based on above findings of the relation between astrocytic calcium dynamics and anxiety-like states, we employed a chemogenetic approach to validate this effect (Fig. 3H). In the open filed test (OFT), mice under with chemogenetic activation of PAG astrocytes exhibited a reduction in both the number of entries into the center zone (1.88 ± 0.51 vs. 33.50 ± 4.97 of the mCherry group, t (14) = 5.915, *p* < 0.0001) and time spent in the center zone (3.61 ± 1.58 vs. 66.28 ± 8.55 of the mCherry group, t (14) = 6.737, *p* < 0.0001; Fig. 3I-J). Similarly, in the EPM test, these mice displayed a decrease in the number of entries into the open arms (1.13 ± 0.28 vs. 3.5 ± 0.43 of the mCherry group, t (14) = 4.326, *p* = 0.0007) and total time spent in the open arms (8.61 ± 3.32 vs. 47.45 ± 8.37 of the mCherry group, t (14) = 4.036, *p* = 0.0012; Fig. 3K-L). Together, these results recapitulated the behavioral effects of SD and provided direct evidence that PAG astrocyte activation may be a trigger of anxiety-like behaviors.

### Manf-mediated astrocyte-neuron interaction was involved in anxiety-like behaviors induced by sleep deprivation

**Fig. 4:**
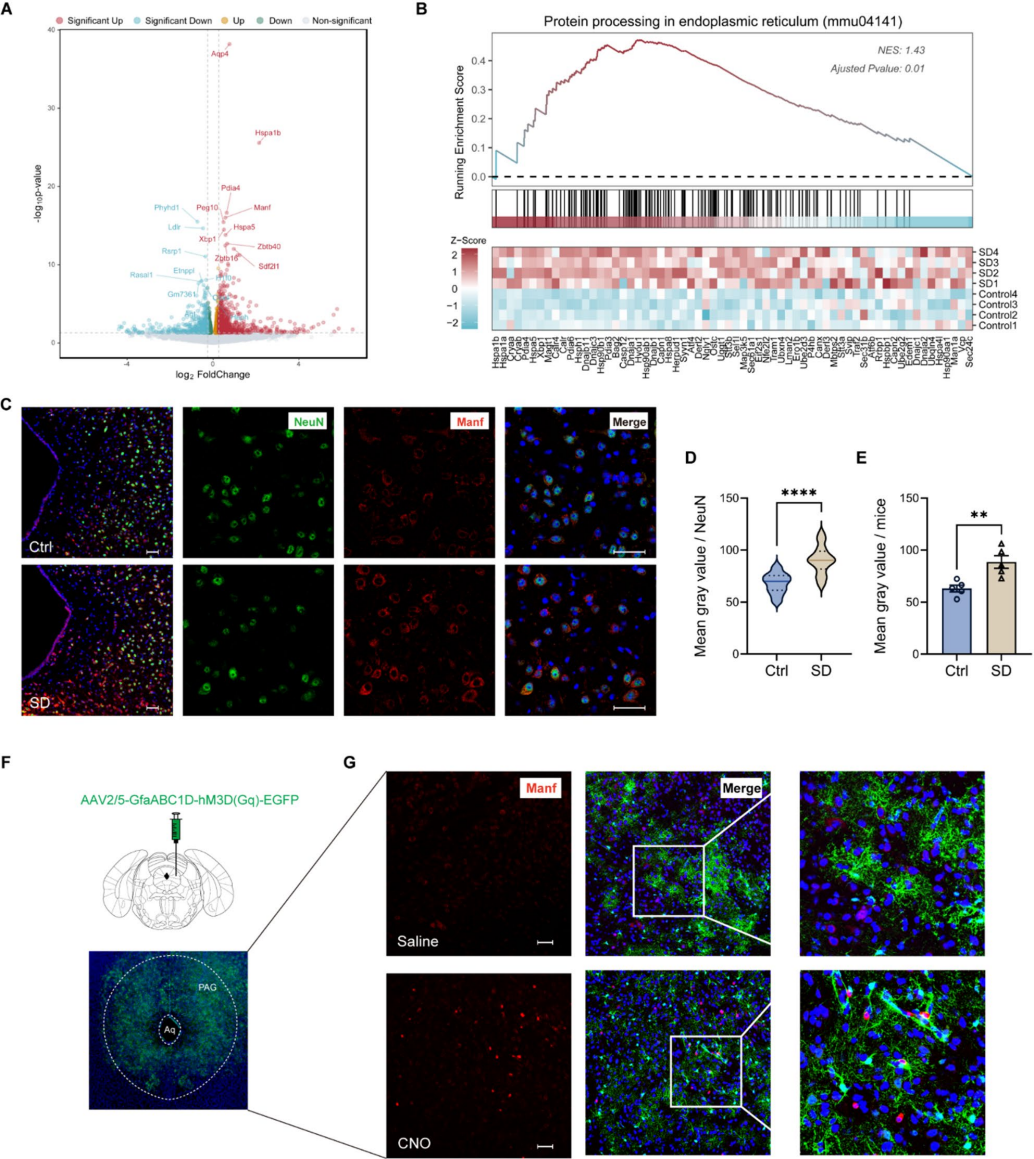
Sleep deprivation increased Manf expression in the periaqueductal gray (PAG). **A** Volcano plot showing differentially expressed genes after sleep deprivation (SD). **B** Gene Set Enrichment Analysis (GSEA) plot showing significant enrichment of pathway after SD; Heatmap showing the expression levels of all genes within this gene set in both groups. **C** Representative immunofluorescence images showing co-localization of Manf (red) with the neuronal marker NeuN (green) in the PAG. Scale bar: 50 μm. **D** Quantification of Manf fluorescence intensity per NeuN. **E** Systemic quantification of Manf fluorescence intensity per mice (*n* = 5). **F** Representative immunofluorescence images showing Manf expression in the PAG; Scale bar: 50 μm. Data were presented as mean ± SEM; **p < 0.01, ****p < 0.0001

To elucidate the molecular mechanisms underlying sleep deprivation (SD)-induced anxiety-like behaviors, we performed bulk RNA sequencing of the periaqueductal gray (PAG) tissue sampled from the control and SD mice. Differential gene expression analysis identified multiple significantly regulated transcripts (Fig. 4A), with the most significant upregulated gene, *Aquaporin-4 (Aqp4)*, encoding the astrocytic water channel aquaporin-4, providing independent molecular validation of astrocytic activation in the PAG following SD. Further, gene ontology (GO) and KEGG pathway analysis of differentially expressed genes showed a significant enrichment of pathways associated with endoplasmic reticulum (ER) protein synthesis and processing (Figure S4A-B). Gene set enrichment analysis (GSEA) further confirmed a strong positive correlation between SD and the ER protein processing pathway (Fig. 4B). SD upregulated multiple genes associated with this pathway, including *Xbp1*, *Pdia4* and *Atf4* (Fig. 4B). Among these, *Manf*, which encodes mesencephalic astrocyte-derived neurotrophic factor, is known to be an ER stress‑responsive protein. Immunofluorescence staining further showed that Manf protein was primarily enriched in neurons, but not astrocytes in PAG; and its neuronal expression was increased (88.61 ± 5.46 vs. 63.07 ± 2.87 of the Ctrl group, t (8) = 3.704, *p* = 0.0060) after SD (Fig. 4C-E). In addition, chemogenetic activation of PAG astrocytes also induced an increase in Manf protein levels in PAG neurons (Fig. 4F-G).

**Fig. 5:**
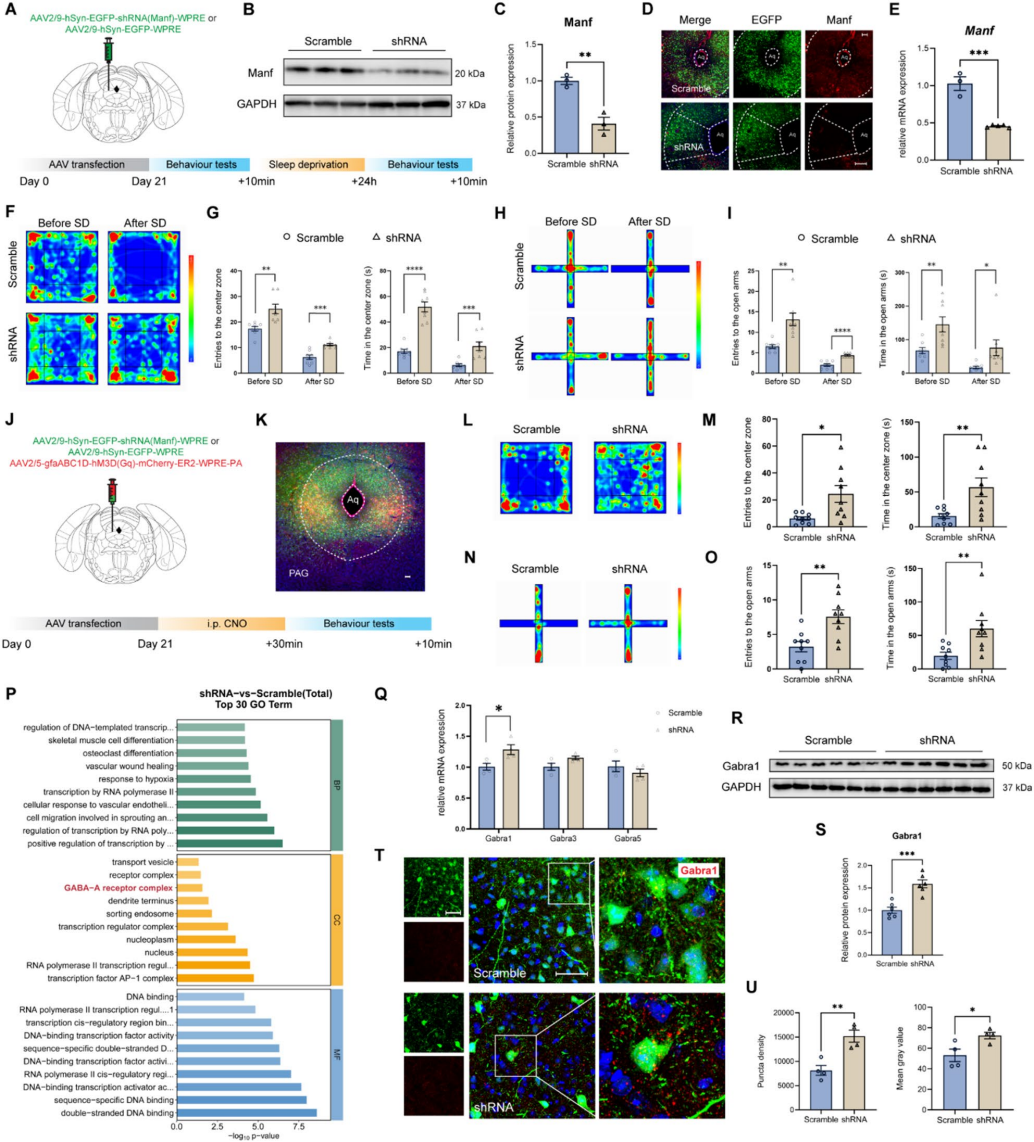
Knockdown of PAG neuronal Manf rescued sleep deprivation-induced anxiety-like behaviors through upregulating GABA_A_ receptor subunit Gabra1. **A** Schematic of knockdown of periaqueductal gray (PAG) neuronal Manf using an AAV vector expressing shRNA. **B** Western blot analysis of Manf protein levels in the PAG following injection of *shRNA Manf* or *shRNA Scramble* viruse*s*. **C** Quantification of Manf protein levels normalized to GAPDH (*n* = 3). **D** Representative immunofluorescence images verifying the knockdown efficiency of Manf in PAG neurons; Scale bar: 50 μm. **E** RT-qPCR analysis of *Manf* mRNA levels in the PAG (*n* = 3 for the control group and *n* = 5 for the knockdown group). **F** Heatmaps of movement tracing in the open field test (OFT) in both groups with or without sleep deprivation (SD). **G** The number of entries into the center zone and the total time spent in the center zone during the OFT (*n* = 8). **H** Heatmaps of movement tracing in the elevated plus maze (EPM) test. **I** The number of entries into the open arms and the total time spent in the open arms during the EPM test (*n* = 8). **J** Schematic of the combined knockdown of PAG neuronal Manf with chemogenetic activation of PAG astrocytes. **K** Representative images confirming the co-transfections of both viruses; Scale bar: 50 μm. **L** Heatmaps of movement tracing in the OFT. **M** The number of entries into the center zone and the total time spent in the center zone (*n* = 9). **N** Heatmaps of movement tracing in the EPM test. **O** The number of entries into the open arms, and the total time spent in the opened arms (*n* = 9). **P** Gene Ontology (GO) showing the differently significant biological processes from the RNA-sequencing data after Manf knockdown. **Q** mRNA expression levels of GABA_A_ receptor subunits *Gabra1*, *Gabra3*, and *Gabra5* in the PAG after Manf knockdown, as validated by qPCR (*n* = 3). **R** Western blot analysis of Gabra1 protein expression levels in the PAG following *Manf* knockdown. **S** Quantification of Gabra1 protein levels normalized to GAPDH (*n* = 6). **T** Representative immunofluorescence images showing Gabra1 expression in PAG neurons after *Manf* knockdown; Scale bar: 50 μm. **U** Quantification of Gabra1 fluorescence intensity in NeuN-positive neurons (*n* = 4). Data were presented as mean ± SEM; *p < 0.05, **p < 0.01, ***p < 0.001, ****p < 0.0001

To determine whether ER stress signaling is functionally linked to the behavioral phenotype, we delivered a short-hairpin RNA (*shRNA*) into an adeno-associated virus (AAV) under a neuronal promoter (hSyn) to achieve neuronal-specific knockdown of *Manf*. The *AAV-shRNA Manf* or a control *AAV-shRNA Scramble* was injected into the PAG (Fig. 5A). Western blot, RT-qPCR and immunohistochemistry confirmed a highly efficient and localized knockdown of *Manf* expression in PAG neurons (Fig. 5B-E). Transcriptomic analysis showed that *Manf* knockdown altered the expression of several ER stress‑associated genes (Figure S4E), indicating that Manf may serve as a key regulatory node in the ER stress network. Furthermore, behavioral tests showed that mice transfected with *AAV-shRNA Manf* have more entries into the center zone (11.29 ± 0.48 vs. 6.25 ± 0.82 of the Scramble group, t (14) = 4.859, *p* = 0.0003) and time spent in the center zone (19.11 ± 2.87 vs. 6.24 ± 1.04 of the Scramble group, t (14) = 4.222, *p* = 0.0009) in the open field test (OFT) compared with scramble group following SD (Fig. 5F-G). Similarly, in the elevated plus maze (EPM) test, *Manf*-knockdown mice entered the open arms more frequently (4.43 ± 0.19 vs. 2.00 ± 0.31 of the Scramble group, t (14) = 6.333, *p* < 0.0001) and spent more time in the open arms (80.14 ± 24.68 vs. 16.43 ± 3.93 of the Scramble group, t (14) = 2.488, *p* = 0.0260) than scramble group mice after SD (Fig. 5H-I).

To further determine whether Manf is required for astrocyte-to-neuron mediated anxiety-like behaviors, we combined neuronal *Manf* knockdown concurrent with chemogenetic activation of PAG astrocytes (Fig. 5J-K). Compared to the scramble group, *Manf*-deficient mice exhibited reduced anxiety with more center-zone entries (24.44 ± 5.91 vs. 6.22 ± 1.16 of the Scramble group, t (16) = 2.852, *p* = 0.0115) and time (56.80 ± 12.48 vs. 15.51 ± 3.35 of the Scramble group, t (16) = 3.013, *p* = 0.0083) in the OFT (Fig. 5L-M), following CNO treatment; and more open- arm entries (7.56 ± 0.93 vs. 3.22 ± 0.70 of the Scramble group, t (16) = 3.509, *p* = 0.0029) and time spent in the open arms (60.13 ± 11.38 vs. 19.58 ± 4.98 of the Scramble group, t (16) = 3.078, *p* = 0.0072) in the EPM test (Fig. 5N-O).

### Astrocyte‑derived glutamate mediated sleep deprivation‑induced Manf upregulation and anxiety‑like behaviors

Astrocytes regulate neuronal activity through the release of gliotransmitters, including glutamate and ATP [29, 30]. We, therefore, investigated whether astrocyte-derived glutamate contributes to sleep deprivation (SD)‑induced anxiety-like behavior. Firstly, we monitored real‑time glutamate dynamics in the periaqueductal gray (PAG) during the EPM test using the glutamate sensor (hSyn‑GluSnFR) expressed in neurons (Figure S5A-B). In control mice, entries into the open arms induced a transient increase in glutamate release. Notably, this open‑arm‑associated glutamate release was further enhanced in SD mice compared to controls (4.43 ± 0.34 vs. 2.09 ± 0.10, t (10) = 3.885, *p* = 0.0030) (Figure S5C-F).

To determine whether astrocytic vesicular release is required for this effect, we selectively inhibited astrocytic exocytosis by expressing tetanus neurotoxin (TeNT) under an astrocyte‑specific promoter (Figure S5G-H). Effective inhibition of astrocytic vesicle fusion was verified by the loss of GluSnFR responses during open‑arm exploration in the EPM test (Figure S5I). Importantly, TeNT‑mediated inhibition of astrocytic glutamate release reversed SD‑induced anxiety‑like behaviors. TeNT‑expressing SD mice exhibited increased center-zone entries (30.75 ± 3.52 vs. 11.75 ± 1.89 of the Ctrl group, t (14) = 4.450, *p* = 0.0005) and time (117.46 ± 19.97 vs. 18.56 ± 3.99, t (14) = 4.544, *p* = 0.0005) in the OFT (Figure S5K‑L) as well as increased open‑arm entries (13.25 ± 1.21 vs. 8.13 ± 0.78, t (14) = 3.333, *p* = 0.0049) and time (22.09 ± 7.33 vs. 2.68 ± 0.84, t (14) = 2.463, *p* = 0.0274) in the EPM test (Figure S5M-N) compared to control mice. Furthermore, TeNT expression reduced the SD‑induced upregulation of neuronal Manf protein (28.297 ± 0.462 vs. 36.008 ± 0.413 of the Ctrl group, t (8) = 11.12, *p* < 0.0001) in the PAG (Figure S5J, V).

To identify the postsynaptic glutamate receptors responsible for these effects, we microinfused either the NMDA receptor antagonist AP5 or the AMPA receptor antagonist CNQX into the PAG after SD (Figure S5O-P). AP5 infusion ameliorated SD‑induced anxiety‑like behaviors, as shown by increased center-zone entries (21.00 ± 1.26 vs. 8.60 ± 1.12 of the Saline group, t (18) = 7.000, *p* < 0.0001) and time (57.29 ± 5.63 vs. 16.34 ± 3.26 of the Saline group, t (18) = 5.976, *p* < 0.0001) in the OFT (Figure S5Q-R) and increased open‑arm entries (4.90 ± 0.62 vs. 1.10 ± 0.26 of the Saline group, t (18) = 5.327, *p* < 0.0001) and time (38.49 ± 4.12 vs. 4.75 ± 1.52 of the Saline group, t (18) = 7.293, *p* < 0.0001) in the EPM test (Figure S5S-T). AP5 also normalized the SD‑induced increase in neuronal Manf expression (26.468 ± 0.593 vs. 36.887 ± 0.778 of the Saline group, t (8) = 9.449, *p* < 0.0001) (Figure S5U-W). In contrast, CNQX infusion had no significant effect on either behavior (center-zone entries: 8.40 ± 2.00 vs. 8.60 ± 1.12 of the Saline group, t (18) = 0.0828, *p* = 0.9349; center-zone time: 13.65 ± 4.39 vs. 16.34 ± 3.26 of the Saline group, t (18) = 0.4667, *p* = 0.6463; open-arm entries: 1.50 ± 0.16 vs. 1.10 ± 0.26 of the Saline group, t (18) = 1.238, *p* = 0.2317; open-arm time: 6.87 ± 1.24 vs. 4.75 ± 1.52 of the Saline group, t (18) = 1.024, *p* = 0.3194) or Manf expression (34.660 ± 0.975 vs. 36.887 ± 0.778 of the Saline group, t (8) = 1.588, *p* = 0.1509), indicating that NMDA receptors, rather than AMPA receptors, primarily mediate astrocyte‑derived glutamate signaling in this pathological process.

### Manf-regulated GABA_A_ receptor subunit alpha1 expression mediated PAG neuronal excitability

To uncover the molecular mechanism through which neuronal Manf influences anxiety-like behaviors, RNA sequencing on periaqueductal gray (PAG) tissue from mice transfected with *AAV-shRNA Manf* or *AAV-shRNA Scramble* identified 303 differentially expressed genes (Figure S4C). Pathway enrichment analysis pointed to a significant effect on GABAergic signaling, specifically implicating the GABA_A_ receptor pathway (Fig. 5P; Figure S4D). We further used RT-qPCR to assess the expression of key synaptic GABA_A_ receptor subunits, and found that gamma-aminobutyric acid type A receptor subunit alpha1 (*Gabra1*) mRNA was upregulated following *Manf* knockdown (1.28 ± 0.07 vs. 1.01 ± 0.05 of the Scramble group, t (6) = 2.887, *p* = 0.0278), while other subunits (*Gabra3*,* Gabra5*) remained unchanged (Fig. 5Q). This increase was confirmed at the protein level by western blot analysis, which showed upregulation of Gabra1 in PAG in *Manf*-knockdown mice (1.59 ± 0.08 vs. 1.00 ± 0.06 of the Scramble group, t (10) = 5.298, *p* = 0.0003; Fig. 5R-S). Furthermore, immunofluorescence staining also demonstrated an increase in the puncta density (15193.00 ± 1067.78 vs. 8143.50 ± 866.95 of the Scramble group, t (6) = 4.439, *p* = 0.0044) and intensity (72.38 ± 2.73 vs. 53.16 ± 5.30 of the Scramble group, t (6) = 2.793, *p* = 0.0315) of Gabra1 clusters on neuronal membranes following *Manf* knockdown (Fig. 5T-U).

### PAG^GABA^-ACC circuit regulated anxiety-like behaviors induced by sleep deprivation

**Fig. 6:**
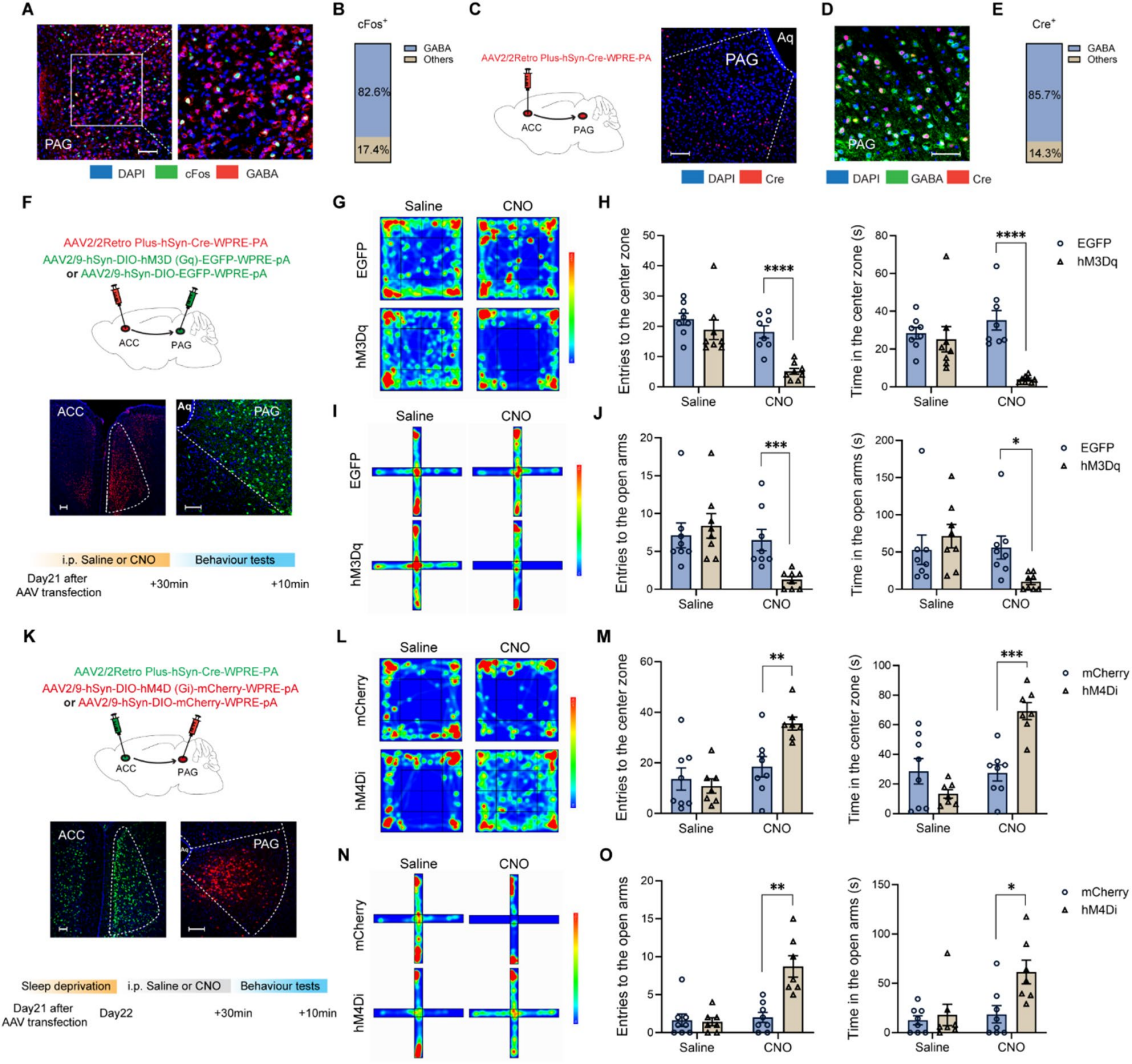
The PAG^GABA^-ACC circuit was involved in SD-induced anxiety-like behaviors. **A** Representative immunofluorescence images showing co-localization of c-Fos and GABA in the periaqueductal gray (PAG) following sleep deprivation (SD); Scale bar: 50 μm. **B** Quantification of the percentage co-localization of c-Fos and GABA after SD. **C** Schematic of the retrograde tracing from the anterior cingulate cortex (ACC) to the PAG. Representative image of retrograde-labeled neurons in the PAG; Scale bar: 50 μm. **D** Representative immunofluorescence images showing co-localization of the retrograde tracer and GABA in the PAG. **E** Quantification of the percentage of retrograde-labeled neurons in the PAG. **F** Schematic of the viral injections for chemogenetic activation of the PAG^GABA^-ACC pathway. **G** Heatmaps of movement tracing in the open field test (OFT) following chemogenetic activation of the PAG^GABA^-ACC pathway. **H** The number of entries into the center zone, and the total time spent in the center zone during the OFT (*n* = 8). **I** Heatmaps of movement tracing in the elevated plus maze (EPM) test following chemogenetic activation. **J** The number of entries into the open arms, and the total time spent in the open arms during the EPM testing (*n* = 8). **K** Schematic of the viral injections for chemogenetic inhibition of the PAG^GABA^-ACC pathway after SD. **L** Heatmaps of movement tracing in the OFT following chemogenetic inhibition of the PAG^GABA^-ACC pathway after SD. **M** The number of entries into the center zone, and the total time spent in the center zone (*n* = 7). **N** Heatmaps of movement tracing in the EPM test following chemogenetic inhibition after SD. **O** The number of entries into the open arms, and the total time spent in the open arms during the EPM test (*n* = 7). Data were presented as mean ± SEM; *p < 0.05, **p < 0.01, ***p < 0.001, ****p < 0.0001

Given that Manf modulates periaqueductal gray (PAG) neuronal excitability through GABA_A_ receptor pathway, we next asked whether this activity underlines anxiety-like behaviors induced by sleep deprivation (SD). We found that SD increased the c-Fos positive cells in the PAG, and 82.6% of these activated neurons were GABAergic (Fig. 6A-B). Since a prominent GABAergic projection from the PAG to the anterior cingulate cortex (ACC) is known to regulate affective behaviors, we hypothesized this pathway mediates SD-induced anxiety. Retrograde tracing from the ACC confirmed that 85.7% of PAG neurons projecting to the ACC are GABAergic (Fig. 6C–E).

Next, a Cre-retrograde virus was injected into the ACC and a Cre-dependent excitatory DREADD virus (AAV-DIO-hM3Dq) into the PAG, allowing selective activation of PAG GABAergic neurons projecting to the ACC (Fig. 6F). Chemogenetic activation of this circuit was sufficient to recapitulate the anxiety-like phenotype, as evidenced by reductions in entries into the center zone (5.13 ± 0.93 vs. 18.13 ± 1.88 of the EGFP group, t (14) = 5.796, *p* < 0.0001) and time spent in the center zone (4.01 ± 0.57 vs. 35.21 ± 4.81 of the EGFP group, t(14) = 6.03, *p* < 0.0001) in the OFT (Fig. 6G and H); similarly, in the EPM test, mice treated with CNO showed fewer open arm entries (1.25 ± 0.39 vs. 6.50 ± 1.31 of the EGFP group, t (14) = 3.594, *p* = 0.0029) and less time spent in the open arms (10.21 ± 3.62 vs. 56.03 ± 14.40 of the EGFP group, t(14) = 2.886, *p* = 0.0120; Fig. 6I and J) than control mice. Conversely, chemogenetic inhibition of PAG-ACC circuit increased center-zone entries (35.57 ± 2.38 vs. 21.00 ± 3.35 of the mCherry group, t (12) = 3.282, *p* = 0.0066) and time spent in the center zone (69.23 ± 5.33 vs. 31.21 ± 4.24 of the mCherry group, t (12) = 5.169, *p* = 0.0002) in the OFT (Fig. 6K-M) as well as increased open-arm entries (8.71 ± 1.30 vs. 2.29 ± 0.63 of the mCherry group, t (12) = 4.108, *p* = 0.0015) and increased time spent in the open arms (61.53 ± 11.15 vs. 21.40 ± 8.89 of the Saline group, t(12) = 2.605, *p* = 0.0230) in the EPM test (Fig. 6N-O) after SD.

### PAG astrocytic activation enhancing GABAergic transmission output to ACC contributed to anxiety-like behaviors induced by sleep deprivation

**Fig. 7:**
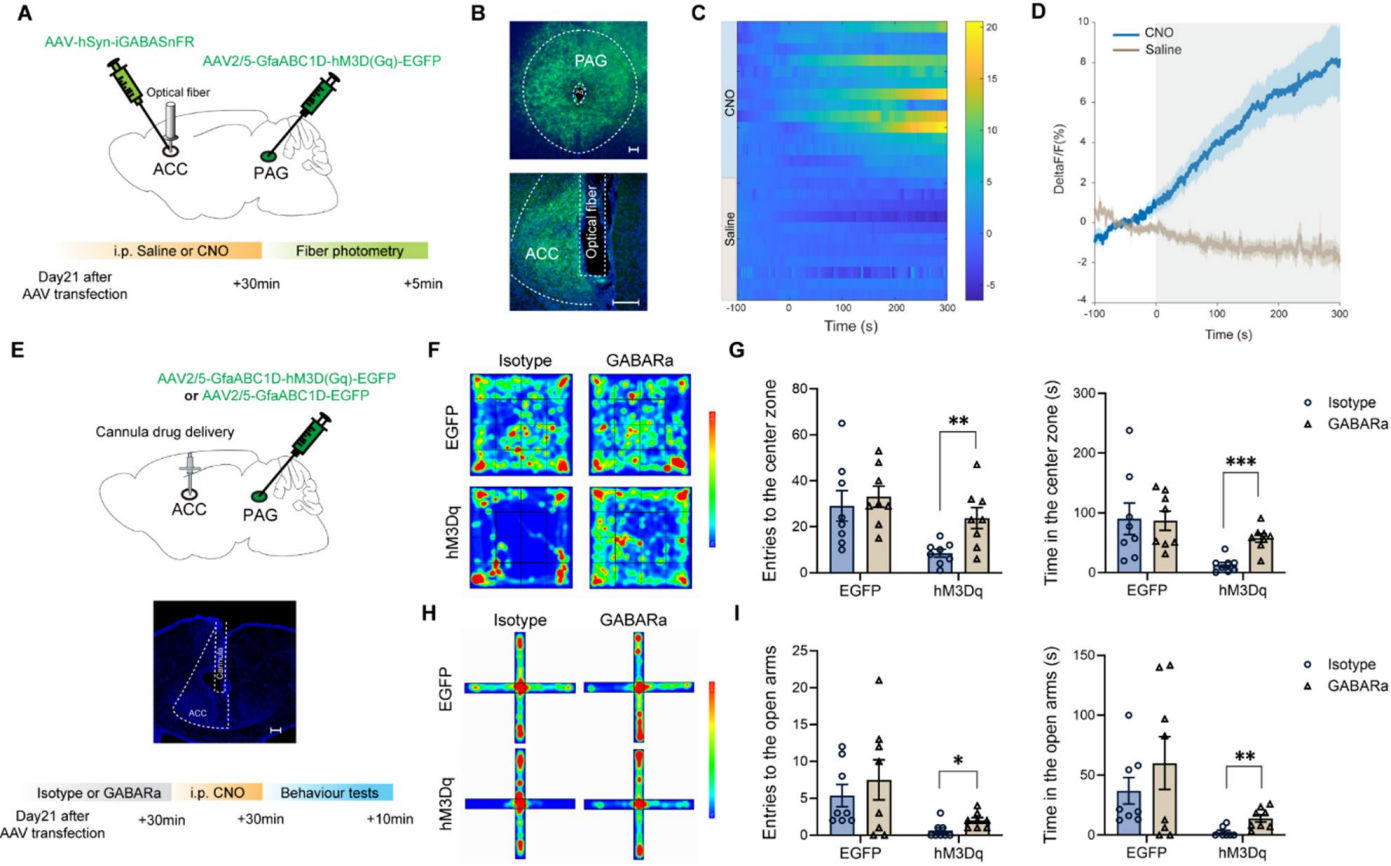
PAG activated astrocyte drove sleep deprivation-induced anxiety-like behaviors through enhancing GABAergic transmission in the ACC. **A** Schematic of virus injections for activating periaqueductal gray (PAG) astrocytes and recording GABA release in the anterior cingulate cortex (ACC). **B** Representative images confirming viral transfections in the PAG and ACC; Scale bars: 50 μm. **C** Heatmap showing GABA dynamic signals in the ACC following intraperitoneal (i.p.) injection of saline or Clonzapine-N-oxide (CNO). **D** Time-course curve showing GABA signal (*ΔF/F*) in the ACC after saline (*n* = 11) or CNO (*n* = 14) administration. **E** Schematic of virus injection for chemogenetic activation of PAG astrocytes combined with micro-infusion of a GABA_A_ receptor antagonist into the ACC. **F** Heatmaps of movement tracing in the open field test (OFT) following chemogenetic activation of PAG astrocytes and micro-infusion of the GABA_A_ receptor antagonist or its isotype control. **G** The number of entries into the center zone, and the total time spent in the center zone during the OFT (*n* = 8). **H** Heatmaps of movement tracing in the elevated plus maze (EPM) test following chemogenetic activation of PAG astrocytes and micro-infusion of the GABA_A_ receptor antagonist or its isotype control. **I** The number of entries into the open arms, and the total time spent in the open arms during the EPM test (*n* = 8). Data were presented as mean ± SEM; *p < 0.05, **p < 0.01, ***p < 0.001

To validate the functional link between periaqueductal gray (PAG) astrocytes and the GABA transmission of PAG-ACC (anterior cingulate cortex) circuit, GABA release in ACC was monitored using fiber photometry, upon chemogenetic PAG astrocytic activation (Fig. 7A and B). After 30 min of CNO administration, mice showed an increase in GABA release in ACC (Fig. 7C and D). Furthermore, local micro-infusion of the GABA_A_ receptor antagonist (GABARa) into the ACC ameliorated anxiety-like behaviors induced by chemogenetic activation of PAG astrocytes, with more center-zone entries (23.75 ± 4.30 vs. 8.50 ± 1.58 of the Isotype group, t (14) = 3.111, *p* = 0.0077) and time spent in the center zone (58.14 ± 6.70 vs. 13.05 ± 4.15 of the Isotype group, t (14) = 5.352, *p* = 0.0001) in the OFT (Fig. 7F and G), as well as increased open-arm entries (2.00 ± 0.35 vs. 0.63 ± 0.35 of the Isotype group, t (14) = 2.582, *p* = 0.0217) and time spent in the open arms (14.10 ± 2.82 vs. 2.48 ± 1.35 of the Isotype group, t (14) = 3.482, *p* = 0.0037) in the EPM test (Fig. 7H and I), compared to isotype control.

## Discussion

**Fig. 8:**
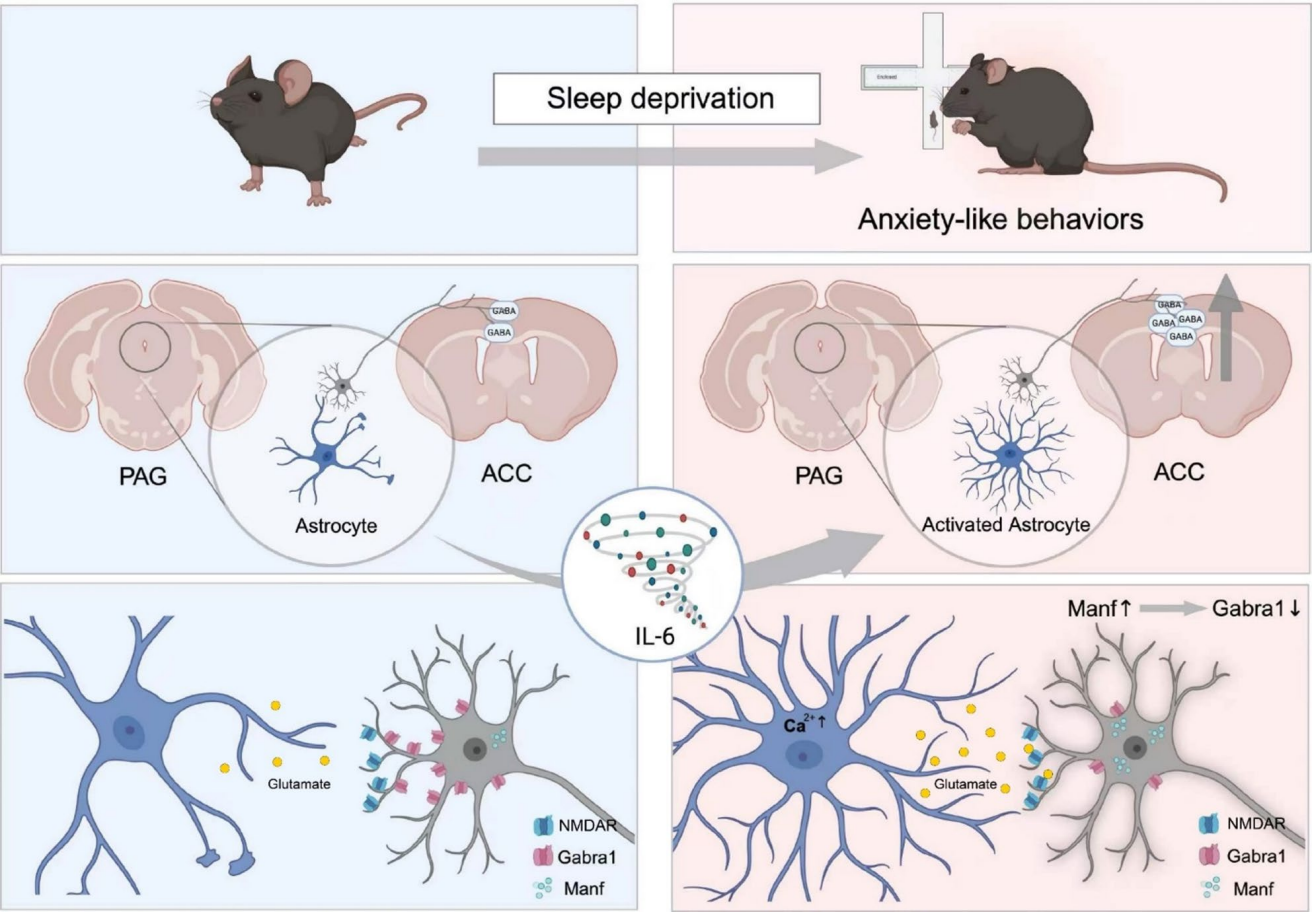
Sleep deprivation induces anxiety-like behaviors through IL-6-driven astrocyte-GABAergic neuron crosstalk in the PAG-ACC circuit. Sleep deprivation (SD) increased IL-6 systemically and in periaqueductal gray (PAG). Elevated IL‑6 induced morphological activation of PAG astrocytes, characterized by increased branch number and length, and enhanced astrocytic calcium activity. Activated astrocytes subsequently released more glutamate, which stimulated postsynaptic NMDA receptors on PAG neurons, induced upregulation of mesencephalic astrocyte‑derived neurotrophic factor (Manf). Increased Manf in turn reduced the expression of the GABA_A_ receptor α1 subunit (Gabra1), thereby disinhibiting GABAergic neurons in the PAG and enhancing GABAergic transmission from the PAG to the anterior cingulate cortex (ACC). This SD-induced circuit-level dysregulation ultimately contributed to anxiety-like behaviors

Our study elucidated the underlying molecular and neural circuit mechanisms of sleep deprivation (SD)-induced anxiety-like behaviors. We demonstrated that SD causes a systemic and midbrain-specific increase of IL-6, thereby activating periaqueductal gray (PAG) astrocytes. This astrocytic response drove the upregulated expression of Manf in neurons, an endoplasmic reticulum (ER) stress-responding protein, which in turn decreased the expression of GABA_A_ α1 receptor expression. The subsequent disinhibition of GABAergic transmission from PAG projections to the anterior cingulate cortex (ACC) induced anxiety-like behaviors. Our work suggests that therapeutic strategies targeting the IL-6-Manf-PAG^GABA^-ACC axis may ameliorate emotional abnormality after SD (Fig. 8).

Acute SD is known to induce behavioral defects. A study in *Drosophila* showed that flies subjected to acute SD on the first day of adult life displayed deficits in short-term memory that persisted for at least six days, indicating that a single acute SD episode can induce long-lasting behavioral changes [31]. Accumulating evidence also indicates that even transient anxiety can reciprocally disrupt sleep homeostasis, establishing a vicious cycle that may ultimately foster chronic comorbidity between sleep disorders and abnormal emotion [32]. Consistent with these findings, the behavioral alterations observed in our study following acute SD may represent early pathophysiological changes that may persist or become exacerbated under chronic conditions. Future studies incorporating different SD durations and longitudinal behavioral assessments are necessary to determine SD-induced temporal progression and related outcomes.

Sleep serves as a fundamental regulator of immune homeostasis, and its disruption exerts profound effects on inflammatory pathways [33, 34]. Acute SD acts as a potent physiological stressor, triggering a systemic inflammatory response including increased release of pro-inflammatory cytokines such as IL-6 and TNF-α [19, 35]. This aligns with our finding that SD increases serum IL-6 in both peripheral and central nervous system. The mechanisms underlying this response may involve the dysregulation of two major effector systems of the hypothalamic-pituitary-adrenal (HPA) axis and the sympathetic nervous system [36, 37]. Activation of β-adrenergic signaling leads to enhanced inflammatory gene expression and cytokine production [38]. Furthermore, sleep loss amplifies innate immune reactivity, as evidenced by increased TLR-4 activity and subsequent cytokine release following partial sleep deprivation [39]. This systemic inflammatory state is not an epiphenomenon but contributes critically to a range of health problems such as metabolic and cardiovascular disorders associated with chronic sleep disturbance [6, 19].

The communication between peripheral inflammation and brain function represents a central focus of neuroimmunology. Pro-inflammatory mediators such as IL-6 enter the central nervous system through multiple routes, including humoral pathways via circumventricular organs, neural afferents such as the vagus nerve, and saturable transport across the blood-brain barrier [40, 41]. Our study provided direct evidence that IL-6 signaling in the PAG is critically involved in sleep deprivation-induced anxiety-like behaviors, highlighting IL-6 as a key mediator led to behavioral alterations. Microglial activation, particularly in the hippocampus, has been shown to induce synaptic pruning and disrupt neurogenesis, contributing to cognitive deficits following sleep disturbance [42]. We found that astrocytes in the PAG are activated by IL-6 and play an indispensable role in anxiety modulation. This astrocytic activation, triggered by inflammatory signals, represented a novel mechanism through which peripheral immune challenges disrupt brain homeostasis leading to anxiety.

Beyond their traditional supportive roles, astrocytes are now recognized as active participants in information processing and behavioral modulation [43]. They respond to a plethora of neuromodulators and neurotransmitters (e.g., glutamate, GABA, adenosine), and in turn, release gliotransmitters (e.g., ATP, glutamate, D-serine) that can modulate synaptic strength, neuronal excitability, and network synchronization [29, 30]. Astrocyte-neuron metabolic coupling, notably through lactate shuttle systems, has been shown to be essential for long-term memory formation [44]. Extending this concept of active astrocytic communication, our study identified mesencephalic astrocyte-derived neurotrophic factor (Manf) as a pivotal messenger in astrocyte-to-neuron communication following sleep deprivation. Although Manf is known for its neuroprotective properties during endoplasmic reticulum (ER) stress response and has emerging link to synaptic function [45, 46], its role in specific behavior‑related neural circuits remains unclear. In Parkinson’s disease, Manf supports dopaminergic neuron survival [47]; Conversely, emerging evidence also indicates that Manf promotes ER stress and contribute to synapse loss in Alzheimer’s disease [48]. Here, we found that neuronal Manf in the PAG was indispensable for astrocyte-mediated anxiety after sleep deprivation. Knockdown of *Manf* specifically downregulated the GABA_A_ receptor α1 (Gabra1), a key determinant of receptor kinetics in synapse. This Manf-dependent Gabra1 downregulation decreased the postsynaptic inhibitory currents (IPSCs) [49], and increased activity of PAG GABAergic neurons, thereby indicating a novel mechanism of SD-induced anxiety reported herein.

GABAergic neurons in the PAG have been implicated not only in sleep-wake regulation but also in the modulation of negative emotional states [15, 18]. Sleep deprivation is known to enhance the activity of these GABAergic neurons, suggesting their potential role in mediating emotional disturbances under conditions of sleep disorders. These neurons project broadly to various brain regions, with the anterior cingulate cortex (ACC) representing as a major downstream target [50]. The ACC is well established as a key node in emotional processing, and its dysfunction was reported to link to anxiety and depression [51]. In this study, we established a causal role of this pathway by demonstrating that chemogenetic activation of the PAG^GABA^-ACC circuit was sufficient to induce anxiety-like behaviors, whereas its inhibition prevented the development of anxiety-like behaviors following SD. Our findings thus delineate a previously unrecognized pathway from PAG GABAergic neurons to the ACC that underlies SD-induced anxiety.

Furthermore, in the lateral habenula (LHb)-locus coeruleus (LC) circuit, astrocyte-neuron signaling during stress exposure critically regulates depressive-like behaviors, highlighting the region-specific roles of astrocytes in emotional pathology [52]. Beyond psychiatric conditions, dysregulated astrocyte-neuron interactions also contribute to the progression of neurodegenerative disorders including Alzheimer’s and Parkinson’s diseases, underscoring their broad involvement in neuronal pathophysiology [53–55]. Our findings that PAG astrocyte activation is both necessary and sufficient for anxiety behavioral development underscored their critical role in emotional control. The specific enhancement of GABAergic transmission in the ACC upon astrocyte activation revealed a mechanism for how astrocytes dynamically modulate the output of a defined anxiolytic/anxiogenic circuit. This positioned astrocytes not as passive supporters, but as dynamic computational elements that integrate inflammatory and neuronal signals to reshape behavioral outcomes.

Our study has several limitations. Firstly, this study was primarily focused on male mice. Although female mice exhibited broadly comparable behavioral changes to sleep deprivation (SD) [23], sex-dependent differences of our findings reported here are unknown and subjected to further study. Secondly, the rotating rod apparatus-based SD model, although widely used for decades, may introduce concurrent stress effects [56]. Although EEG/EMG recordings confirmed significant sleep disruption in our paradigm, the contribution of stress cannot be completely excluded. Nevetheless, compared with other SD methods, e.g., the modified-multiple-platforms-over-water model, the rotating rod paradigm is generally considered to be less stressful, as it does not significantly increase serum stress hormones such as corticosterone, and maintains normal locomotor activity following a 24-h SD [57]. However, future studies employing alternative SD approaches with minimal stress exposure is still needed to further validate the findings reported in our current work.

## Conclusion

In summary, we demonstrated that sleep deprivation triggers a specific increase in IL-6 both systemically and within the midbrain, which activates astrocytes in the periaqueductal gray (PAG). These activated astrocytes released glutamate, which acted on postsynaptic NMDA receptors to upregulate neuronal mesencephalic astrocyte-derived neurotrophic factor (Manf) expression in the PAG. Increased Manf in turn downregulated GABA_A_ receptor α1 subunit (Gabra1) expression, leading to disinhibition of GABAergic projections from the PAG to the anterior cingulate cortex (ACC) and ultimately driving anxiety‑like behaviors. We established that this IL-6-astrocyte-Manf-PAG^GABA^-ACC axis is both necessary and sufficient for sleep deprivation-induced anxiety-like behaviors, not only linking neuroinflammation to negative emotion but also identifying potential therapeutic targets along with signaling cascade for the treatment of sleep deprivation-related affective disorders.

## Supplementary Information

Supplementary Material 1.

Supplementary Material 2.

## Data Availability

All data generated or analyzed during this study are included in this published article (and its supplementary information files). Source data were provided in this paper and are available upon reasonable request to the corresponding authors.

## Abbreviations

SD: Sleep deprivation
PAG: Periaqueductal gray
ACC: Anterior cingulate cortex
IL-1β: Interleukin-1 beta
IL-6: Interleukin-6
TNF-α: Tumor necrosis factor-alpha
MANF: Mesencephalic astrocyte-derived neurotrophic factor
REM: Rapid eye movement
NREM: Non-rapid eye movement
EEG: Electroencephalography
EMG: Electromyography
OFT: Open field test
EPM: Elevated plus maze
IL-6Ra: IL-6 receptor antagonist
GFAP: Glial fibrillary acidic protein
AQP4: Aquaporin-4
Iba1: Ionized calcium-binding adapter molecule 1
TeNT: Tetanus neurotoxin
shRNA: Short-hairpin RNA
CNO: Clonzapine N-oxide
Gabra1: Gamma-aminobutyric acid type A receptor subunit alpha1
GABARa: GABA_A_ receptor antagonist
