# Supplementary material for: Sleep deprivation induces anxiety-like behaviors through IL-6 driven astrocyte-GABAergic neuron crosstalk in the PAG-ACC circuit
Source: J Neuroinflammation. 2026 May 22;23:250. doi: 10.1186/s12974-026-03879-z (PMC13374079; doi:10.1186/s12974-026-03879-z)
Supplement: Supplementary file 1 — Supplementary Material 1. [file 12974_2026_3879_MOESM1_ESM.docx]

**Xu X. et al. Sleep deprivation induces anxiety-like behaviors through IL-6 driven astrocyte-GABAergic neuron crosstalk in the PAG-ACC circuit**

**Supplemental Tables and Figures**

**Table S1. The primer sequences eused.**

| Gene | Forward | Reverse |
| --- | --- | --- |
| *IL-1β* | ATGCCACCTTTTGACAGTGATG | TGTGCTGCTGCGAGATTTGA |
| *IL-6* | CCCCAATTTCCAATGCTCTCCT | GATGGTCTTGGTCCTTAGCCA |
| *TNF-α* | ATGGCCTCCCTCTCATCAGT | TTTGCTACGACGTGGGCTAC |
| *Manf* | TTTTGCCGTGAAGCAAGAGG | TGGTGGCAGCATCATCTGTG |
| *Gabra1* | CATGACAGTGCTCCGGCTAA | CATGACAGTGCTCCGGCTAA |
| *Gabra3* | TTCCCTGTGCTCTTTGCCAT | TGTTTGCGGATCATGCCCTT |
| *Gabra5* | TTACCAGCTTTGGCCCAGTG | TTGTTGAGAGGGAGGCGTTG |
| *GAPDH* | AGGTCGGTGTGAACGGATTTG | TGTAGACCATGTAGTTGAGGTCA |

**Table S2. Details of recombinant adeno-associated viruses (rAAVs) used.**

| Virus strains | Source | Identifier |
| --- | --- | --- |
| AAV2/5-GfaABC1D-jGCaMP7b-WPRE-pA | BrainCase | BC-1341 |
| AAV2/5-GfaABC1D-hM3D(Gq)-mCherry-ER2-WPRE-pA | Taitool | S0483-5 |
| AAV2/1-hSyn-EGFP-pP2A-Cre-WPRE-pA | OBiO | H4942 |
| AAV2/2-hSyn-Cre-WPRE-pA | Taitool | S0278-2-RP |
| AAV2/9-hSyn-DIO-hM3D (Gq)-eGFP-WPRE-pA | Taitool | S0260-9 |
| AAV2/9-hSyn-DIO-hM4D(Gi)-mCherry-WPRE-pA | Taitool | S0193-9 |
| AAV2/5-GfaABC1D-EGFP-P2A-hM3D(Gq)-WPRE-pA | BrainCase | BC-2830 |
| AAV2/9-hSyn-iGABASnFR-WPRE-pA | OBiO | H15052 |
| AAV2/9-hSyn-EGFP-3xFLAG-miR30shRNA(NC)-WPRE-pA | OBiO |  |
| AAV2/9-hSyn-EGFP-3xFLAG-miR30shRNA(Manf)-WPRE-pA | OBiO |  |
| AAV2/9-hSyn-iGluSnFR | BrainCase | BC-0320 |
| AAV2/5-GfaABC1D-mScarlet-F2A-TeNT | BrainCase | BC-4686 |


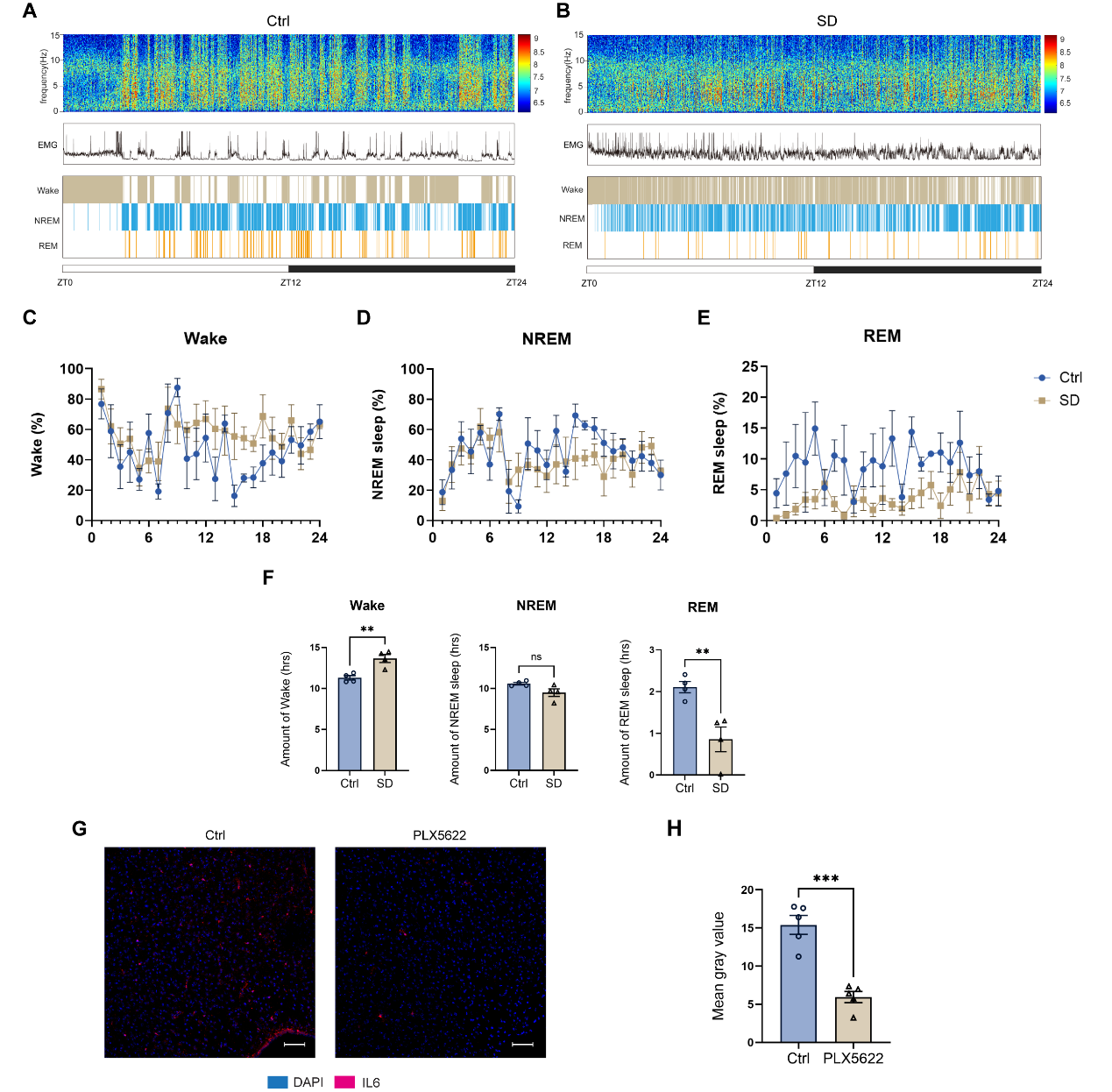


**Figure S1.** **Sleep deprivation reduced REM sleep and induced microglia-dependent IL-6 expression in the midbrain. A-B** Representative 24-hour electroencephalogram (EEG) time-frequency heatmaps, electromyogram (EMG) traces, and corresponding hypnogram illustrating sleep architecture in mice. **C-E** Time course of the percentage of time spent in wake, non-rapid eye movement (NREM), and rapid eye movement (REM) sleep across the 24-hour sleep-deprived period. **F** Quantitative analysis of the total duration of wake, NREM, and REM sleep stages within 24 hours (n=4). G Representative immunofluorescence images showing IL‑6 in the midbrain of control diet‑fed and PLX5622‑treated mice after sleep deprivation (SD). Scale bar: 50 µm. **H** Quantification of IL‑6 fluorescence intensity in the midbrain of control diet‑fed and PLX5622‑treated mice after sleep deprivation (SD) (n=5). Data are presented as mean ± SEM; ***p* < 0.01, ****p* < 0.001.


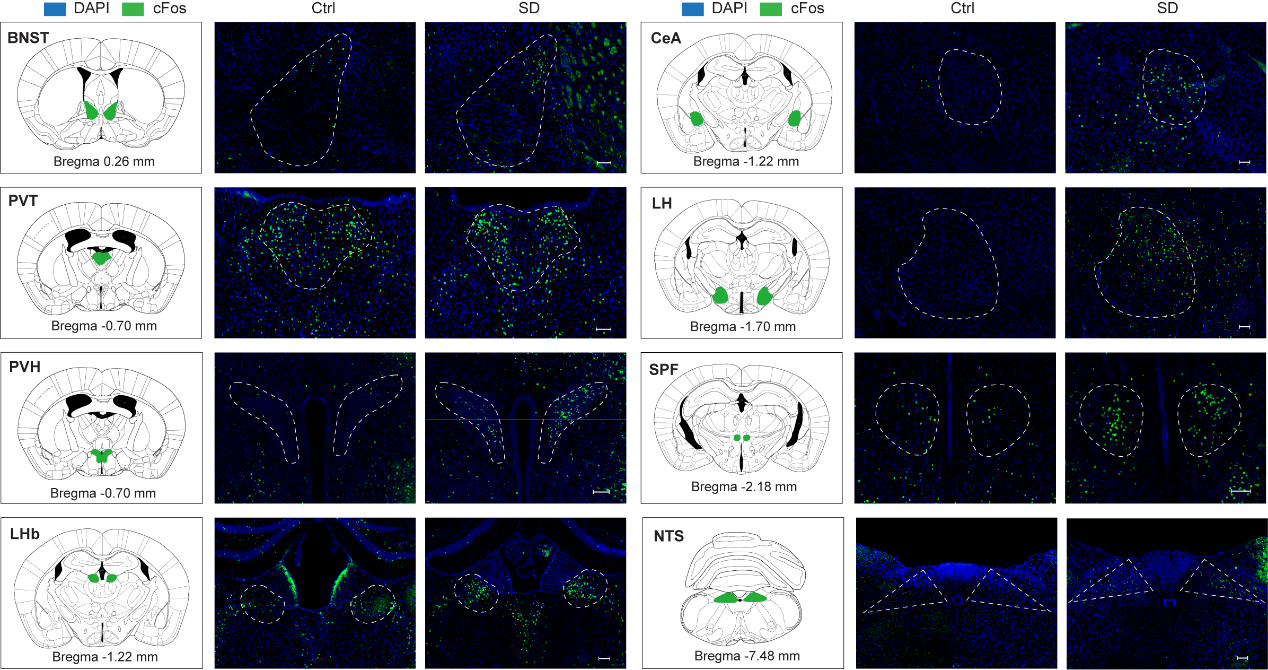


**Figure S2. Representative immunofluorescence images of c-Fos expression.** Representative immunofluorescence images showing c-Fos expression through the whole brain in the control group and the sleep deprivation (SD) group. Scale bar: 100 µm.


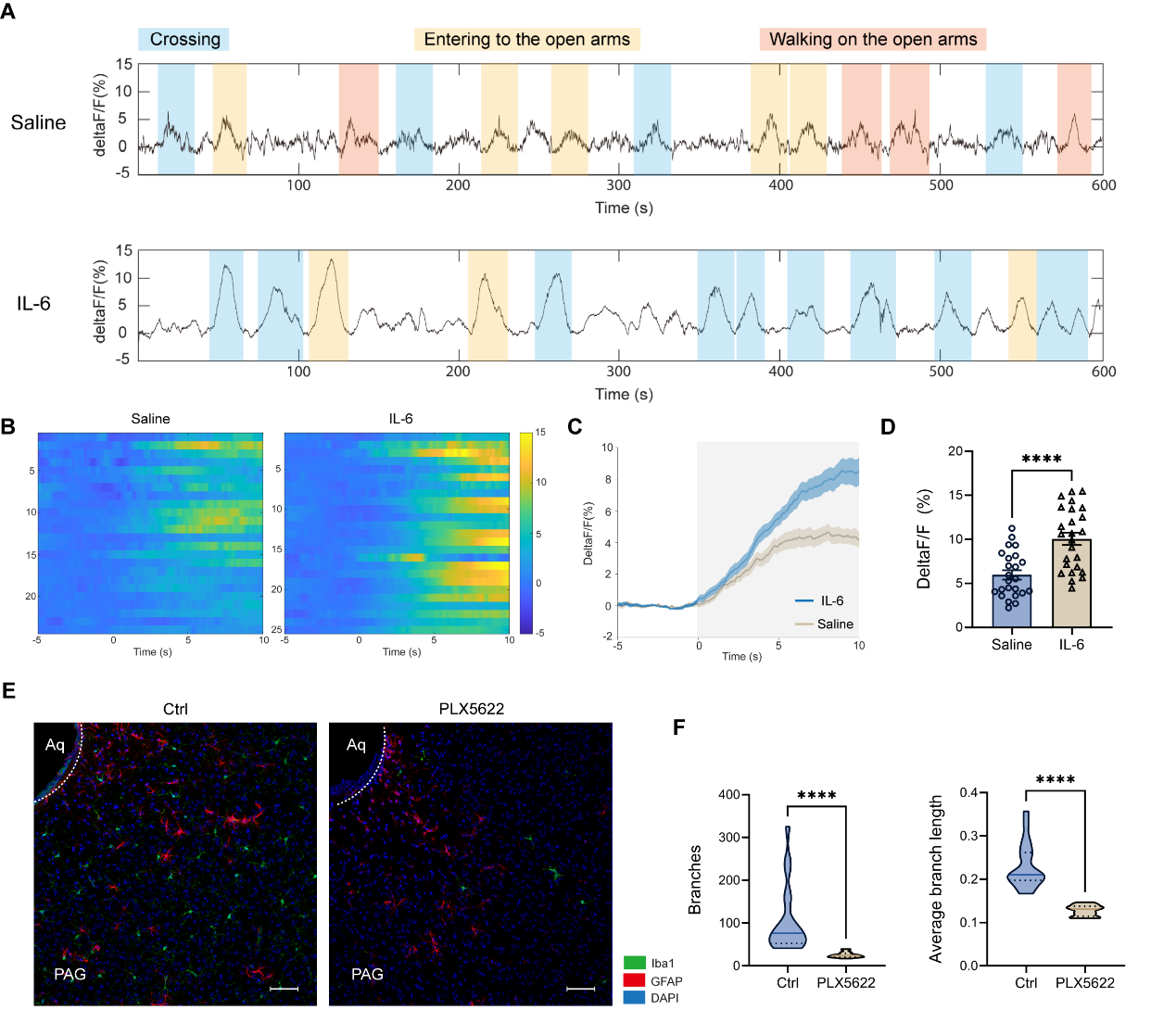


**Figure S3.** **IL-6-inudced astrocyte activation contributed to anxiety-like behaviors induced by sleep deprivation. A** Representative traces of astrocytic calcium activity in the periaqueductal gray (PAG) during the elevated plus maze (EPM) test, following intravenous injection of saline or IL-6. **B** Heatmap of astrocytic calcium signals during the EPM test, aligned to entries into the open arms, after saline or IL-6 injection. **C** Time-course curve of the average astrocytic calcium activity in the PAG during the entries into the open arms in EPM test after saline or IL-6 injection. **D** Quantification of the peak *ΔF/F* of astrocytic calcium signals in the PAG during the entries into the open arms in EPM test following saline or IL-6 injection. **E** Representative immunofluorescence images of GFAP (astrocyte marker) in the PAG of control diet‑fed and PLX5622‑treated mice following sleep deprivation (SD). Scale bar: 50 µm. **F** Quantitative analysis of astrocyte branch number and average branch length in the PAG under the conditions described in (E). Data are presented as mean ± SEM; *****p* < 0.0001.


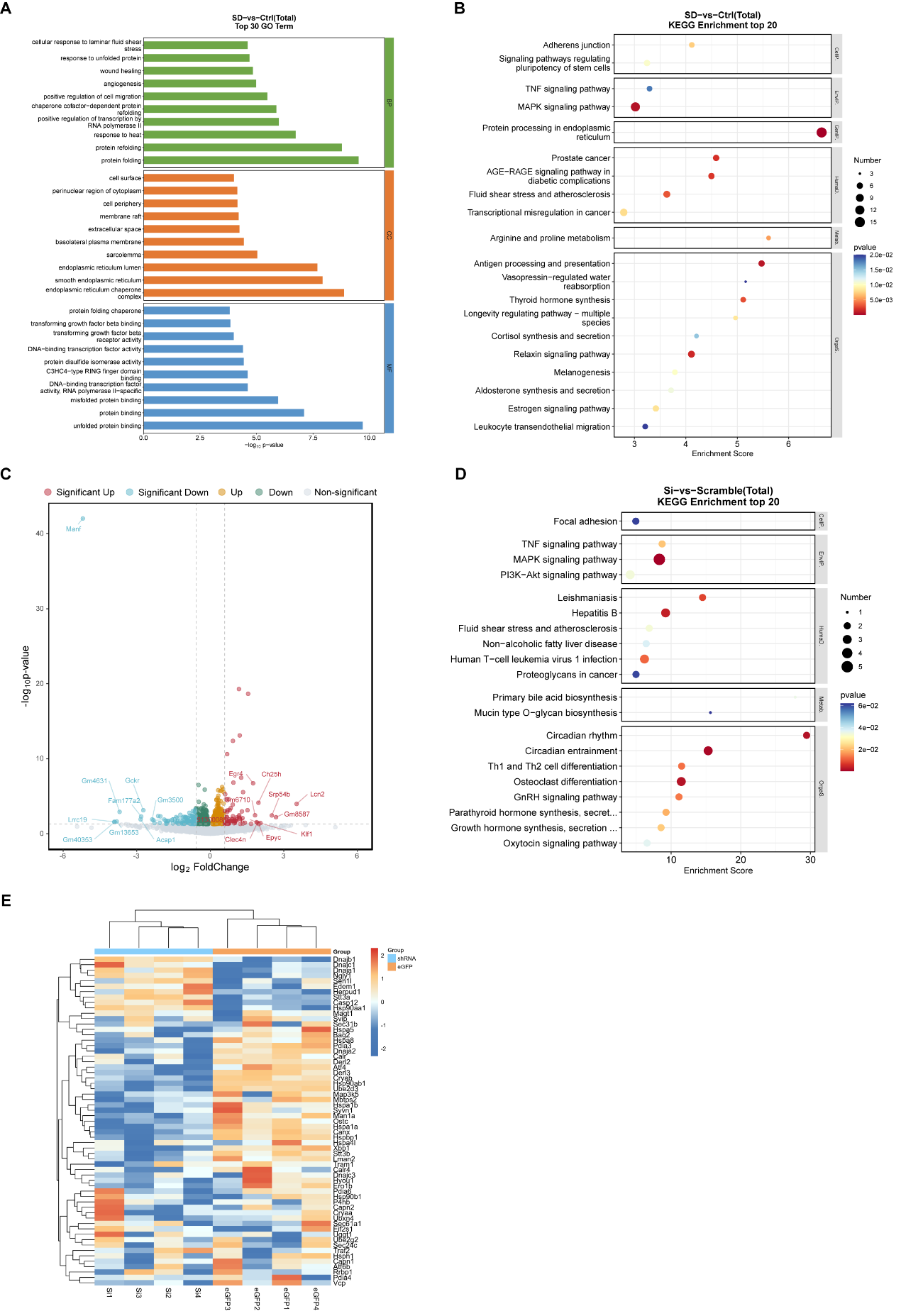


**Figure S4. Transcriptomic analyses of the PAG after sleep deprivation and following *Manf* knockdown. A** Gene Ontology (GO) enrichment analysis of biological processes for differentially expressed genes in the periaqueductal gray (PAG) following sleep deprivation (SD). **B** Kyoto Encyclopedia of Genes and Genomes (KEGG) pathway enrichment analysis of differentially expressed genes in the PAG following SD. **C** Volcano plot displaying differentially expressed genes in the PAG after *Manf* knockdown compared to the control group. **D** KEGG pathway enrichment analysis of differentially expressed genes following *Manf* knockdown in the PAG. **E** Heatmap of ER stress‑related gene expression following *Manf* knockdown in the PAG.

**
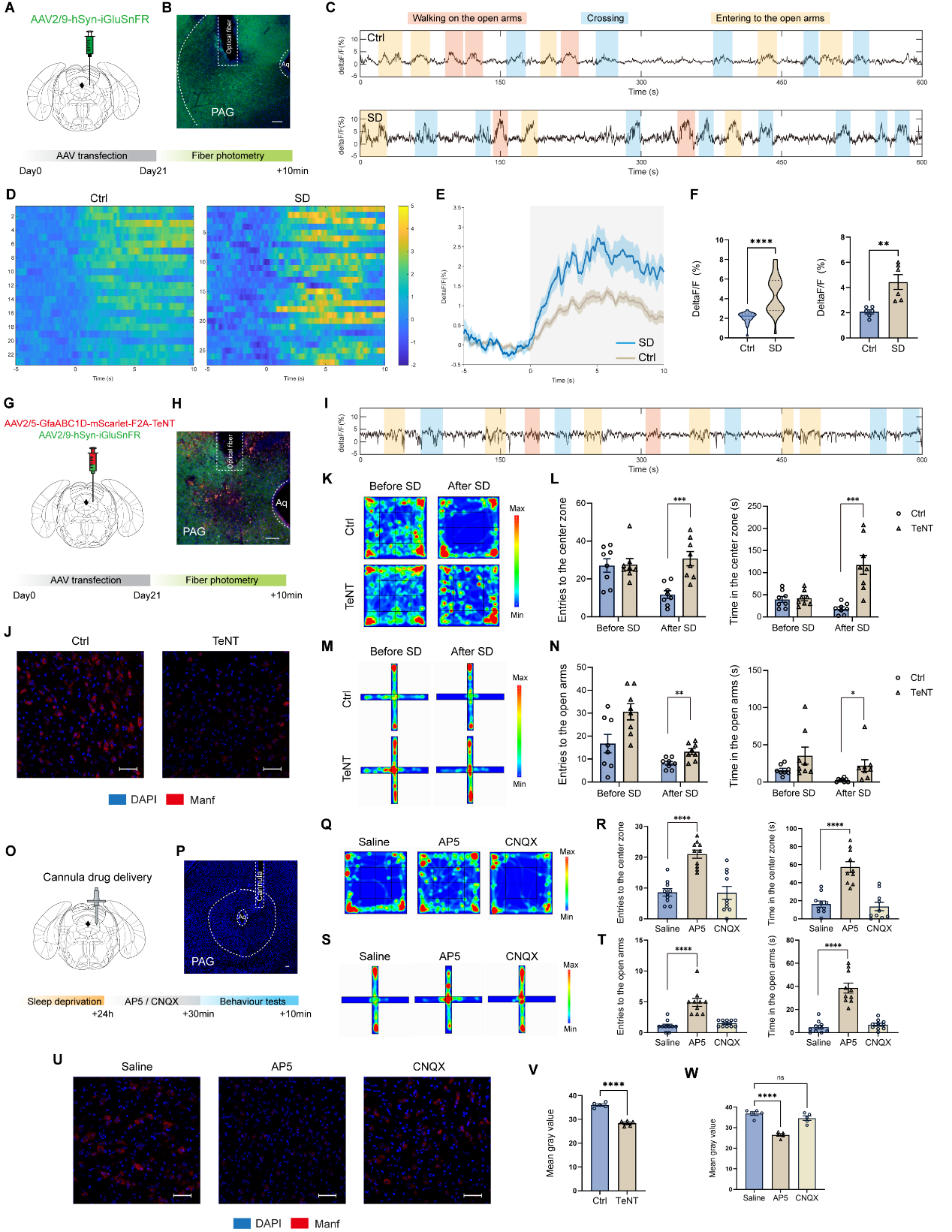
**

**Figure S5. Astrocyte‑derived glutamate mediated sleep deprivation‑induced Manf upregulation and anxiety‑like behaviors. A** Schematic of the neuron‑specific glutamate sensor (hSyn‑iGluSnFR) expressed in the periaqueductal gray (PAG) for fiber photometry recordings. **B** Representative image verifying the viral expression and fiber placement in the PAG. Scale bar: 50 µm. **C** Representative traces of glutamate signals in the PAG during the elevated plus maze (EPM) test in control and sleep deprivation (SD) mice. Shaded areas indicate open‑arm exploration. **D** Heatmap showing glutamate dynamics aligned to open‑arm entries in both groups. **E** Time‑course curve of average glutamate signals during open‑arm exploration. **F** Quantification of peak ΔF/F of glutamate signals during open‑arm entries (n = 6). **G** Schematic of astrocyte‑specific tetanus neurotoxin to block vesicular release co‑expressed with the glutamate sensor in the PAG. **H** Representative image confirming viral expression and fiber placement. Scale bar: 50 µm. **I** Representative traces of glutamate signals in the PAG during the EPM test after TeNT‑mediated inhibition of astrocytic vesicle fusion. **J** Representative immunofluorescence images of Manf in the PAG of control and TeNT‑expressing mice following sleep deprivation (SD). Scale bar: 50 µm. **K** Representative heatmaps of movement tracing in the open field test (OFT) in both groups with or without SD. **L** The number of entries into the center zone and the total time spent in the center zone during the OFT (n=8). **M** Representative heatmaps of movement tracing in the elevated plus maze (EPM) test in both groups with or without SD. **N** The number of entries into the open arms and the total time spent in the open arms during the EPM test (n = 8). **O** Schematic of intracerebral cannula implantation into the PAG for microinfusion of NMDA (AP5) or AMPA (CNQX) receptor antagonists. **P** Representative image verifying cannula placement. Scale bar: 50 µm. **Q** Representative heatmaps of movement tracing in the OFT after AP5, CNQX, or saline infusion in mice following SD. **R** The number of entries into the center zone and the total time spent in the center zone during the OFT (n=10). **S** Representative heatmaps of movement tracing in the EPM test after AP5, CNQX, or saline infusion in mice following SD. **T** The number of entries into the open arms and the total time spent in the open arms during the EPM test (n=10). **U** Representative immunofluorescence images of Manf in the PAG after AP5, CNQX, or saline infusion following SD. Scale bar: 50 µm. **V** Quantification of Manf fluorescence intensity in control and TeNT‑expressing mice following SD (n=5). **W** Quantification of Manf fluorescence intensity after AP5, CNQX, or saline infusion following SD (n=5). Data are presented as mean ± SEM; **p* < 0.05, ***p* < 0.01, ****p* < 0.001, *****p* < 0.0001.
