# Supplementary figures and images for: Sleep deprivation induces anxiety-like behaviors through IL-6 driven astrocyte-GABAergic neuron crosstalk in the PAG-ACC circuit
Source: J Neuroinflammation. 2026 May 22;23:250. doi: 10.1186/s12974-026-03879-z (PMC13374079; doi:10.1186/s12974-026-03879-z)

Figure5 B - Manf 20kDa


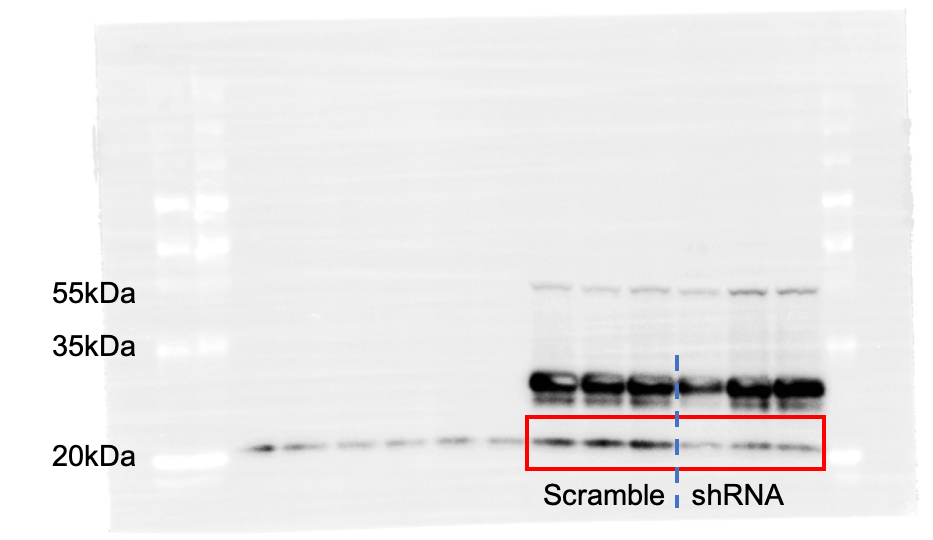


Figure5 B - GAPDH for Manf 37kDa


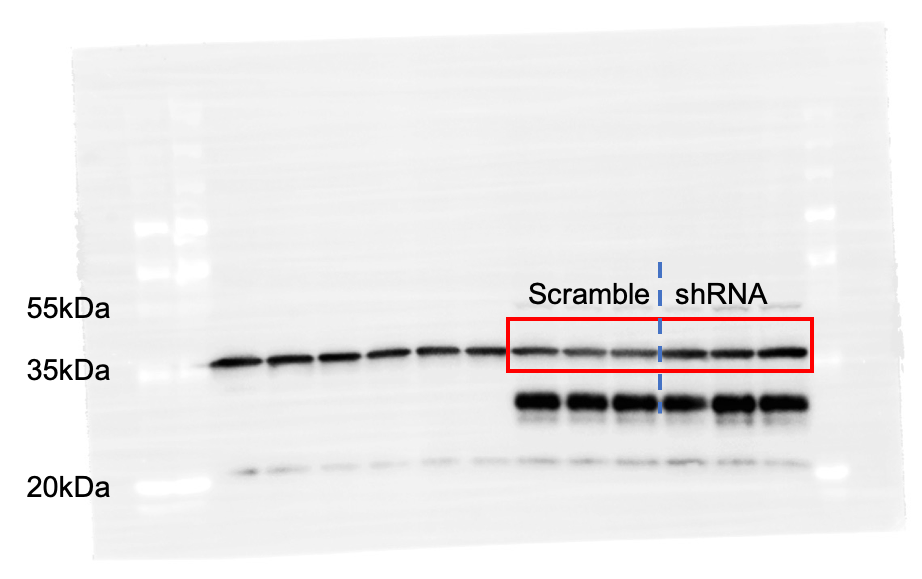


Figure5 R - Gabra1 50kDa


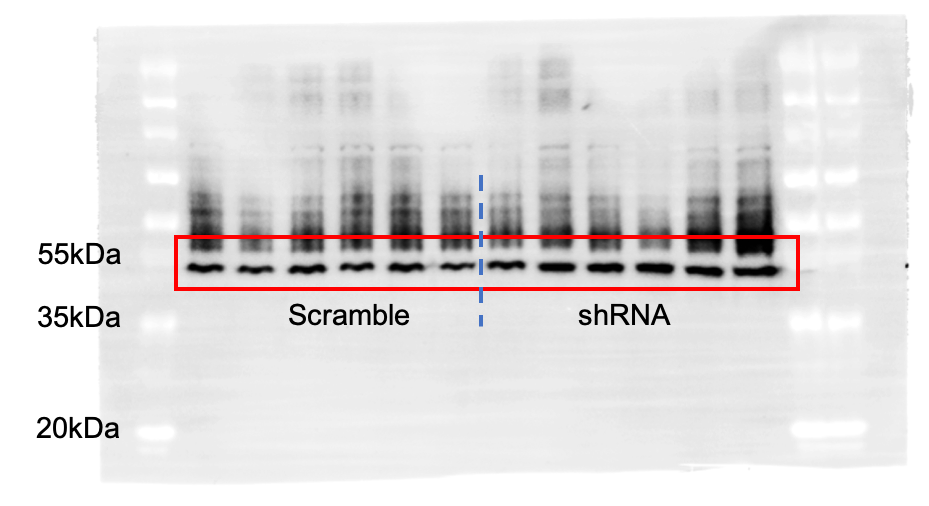


Figure5 R - GAPDH for Gabra1 37kDa


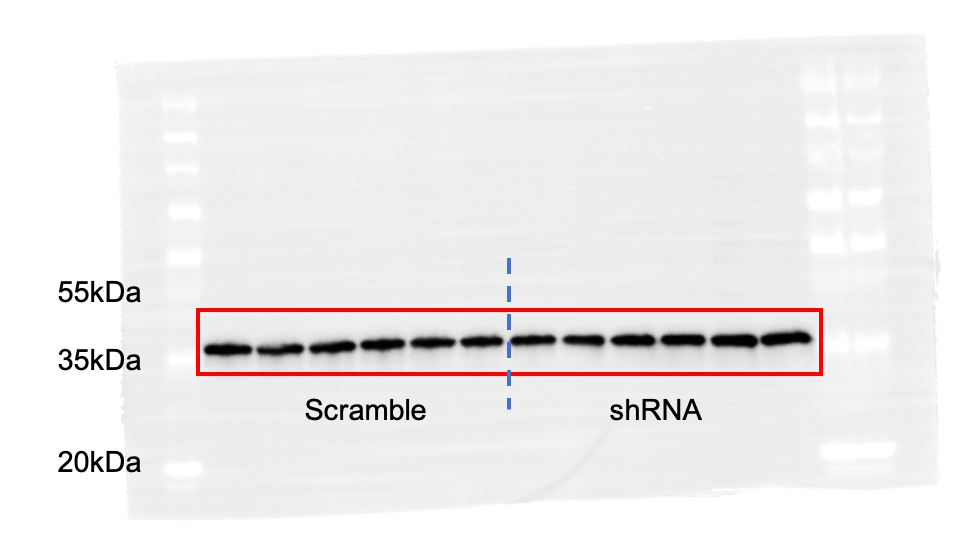

Supplement: Supplementary file 2 — Supplementary Material 2. [file 12974_2026_3879_MOESM2_ESM.docx]
